# Genomics and metabolomics analyses reveal the endophytic fungus *Neocucurbitaria* sp. VM-36 as a potential source of bioactive metabolites against methicillin-resistant *Staphylococcus aureus*

**DOI:** 10.3389/fmicb.2026.1929829

**Published:** 2026-10-15

**Authors:** Xiao Li, Rosario del Carmen Flores-Vallejo, Ting He, Jan Maarten van Dijl, Kristina Haslinger

**Affiliations:** 1 Chemical and Pharmaceutical Biology, University of Groningen, Groningen, Netherlands; 2 University Medical Center Groningen, Department of Medical Microbiology and Infection Prevention, University of Groningen, Groningen, Netherlands; 3 European Institute of Innovation and Technology (EIT), Healthy Ageing PhD School, University Medical Center Groningen, University of Groningen, Groningen, Netherlands

**Keywords:** anti-MRSA, decalin-containing tetramic acids, metabolome, *Neocucurbitaria* sp., whole-genome sequencing

## Abstract

Endophytic fungi associated with medicinal plants represent valuable sources of specialized metabolites with potential biological activities.

**Methods:**

Here, we investigated *Neocucurbitaria* sp. VM-36, an understudied endophyte isolated from the medicinal plant *Vinca minor*, using an integrated genomic, metabolomic, and antibacterial approach.

**Results:**

Whole genome analysis suggests substantial biosynthetic potential for a range of secondary metabolites based on the presence of various biosynthetic gene clusters. Metabolomic profiling revealed a broad chemical repertoire, including metabolites putatively assigned as decalin-containing tetramic acids (DTAs). Among the compounds examined experimentally, compound **1** was structurally identified and exhibited potent antibacterial activity against Gram-positive pathogens, particularly methicillin-resistant *Staphylococcus aureus* (MRSA), with MIC and MBC values of 0.5–1 and 2–4 μg/mL, respectively. Compound **1** displayed rapid bactericidal activity and showed an additive effect in combination with daptomycin against a clinical MRSA isolate (FICI = 0.625). These findings provide evidence for the occurrence of DTA-related metabolites in *Neocucurbitaria* and experimentally demonstrate the antibacterial activity of compound **1**.

**Discussion:**

Collectively, this study provides integrated genomic and metabolomic insights into an endophytic *Neocucurbitaria* strain, expands the known occurrence of DTA-related metabolites in fungi, and supports the potential of *Neocucurbitaria* as a source of antibacterial hit compounds.

## 1 Introduction

Antibiotic resistance has become a global problem threatening human health (Velez and Sloand, 2016). Pathogenic bacteria can cause illnesses by producing toxins that neutralize the human immune defenses and damage healthy tissue, thereby causing harmful infections and posing a serious threat to public health (Wang P. et al., 2024). While antibiotics were initially effective in treating bacterial infections, their widespread use has triggered the rapid transfer of antibiotic resistance genes between bacteria and led to prevalent bacterial resistance against clinically applied drugs (Li et al., 2023). Many bacterial pathogens can nowadays withstand multiple antibacterial agents, making the infections they cause increasingly difficult to treat (Li et al., 2023). This is particularly relevant to ESKAPE(E) pathogens, which include *Enterococcus faecium*, *Staphylococcus aureus*, *Klebsiella pneumoniae*, *Acinetobacter baumannii*, *Pseudomonas aeruginosa*, *Enterobacter* spp., along with *Escherichia coli.* These bacteria are major drivers of healthcare-associated and biofilm infections, posing severe clinical challenges (Asokan et al., 2026a, 2026b). For instance, methicillin-resistant *S. aureus* strains have acquired the *mecA* gene, which encodes a low-affinity penicillin-binding protein that allows cell wall synthesis to continue despite the presence of *β*-lactams antibiotics (Peacock and Paterson, 2015). The increasing prevalence of multidrug-resistant strains, combined with the slow development of effective antibiotics, has severely limited the available therapeutic options. Therefore, there is a pressing need to discover antibacterial agents.

Endophytic fungi from medicinal plants are rich resources of diverse chemical scaffolds, including polyketides, alkaloids, terpenoids, lactones, anthraquinones, quinones, glycosides, steroids and lignans (Gupta et al., 2020; Wen et al., 2022). Medicinal plants are particularly attractive hosts because they harbor diverse endophytic microorganisms that have co-evolved with their hosts and frequently produce structurally diverse bioactive secondary metabolites. *Vinca minor*, a medicinal plant belonging to the Apocynaceae family, is well known for producing pharmacologically important indole alkaloids and therefore represents a promising reservoir of metabolically versatile endophytic fungi (He et al., 2025). To explore this potential, we previously established an in-house endophytic fungal library from *V. minor* and conducted in-depth genomic and metabolomic investigations of two representative isolates, *Fusarium* sp. VM-40 (He et al., 2023) and *Cosmosporella* sp. VM-42 (He et al., 2025). During antibacterial screening of our fungal collection, the isolate VM-36 was prioritized for further investigation because its crude ethyl acetate (EtOAc) extract consistently exhibited one of the strongest inhibitory activities against the indicator strain *Bacillus subtilis* 168 (Supplementary Figure S1). Furthermore, internal transcribed spacer (ITS) sequencing analysis indicated that the fungus belongs to *Neocucurbitaria*, a little studied genus from the Cucurbitariaceae family.

To date, only approximately 25 publications on this genus can be retrieved from the Web of Science database, with the earliest report published in 2017. Most studies have focused on fungal ecology, with only three have investigated fungal secondary metabolites. These studies reported 17 diterpenoids from the marine fungus *Neocucurbitaria unguis-hominis* FS685, including neocucurbols A-H, neocucurbins A-G, and neocucurins A and B (Hu et al., 2022a, 2022b; Liu et al., 2026). Biological evaluation showed that most of these compounds lacked antibacterial and cytotoxic activities (Hu et al., 2022a, 2022b). Among them, only neocucurin B exhibited moderate antibacterial activity against *Staphylococcus aureus* (MIC = 25 μM), while remaining inactive against *Escherichia coli*, and none of the compounds displayed significant cytotoxicity against the tested human cancer cell lines, including the SF-268 (glioblastoma carcinoma), MCF-7 (breast cancer), HepG-2 and A549 (liver cancer) cell lines (Hu et al., 2022a, 2022b). The limited information currently available on *Neocucurbitaria* species indicates that the secondary metabolic diversity, biosynthetic potential and bioactivities of this genus remain largely unexplored. The pronounced antibacterial activity of isolate VM-36, together with the limited knowledge of the metabolic potential of this genus, prompted us to investigate this strain in greater detail.

To address these knowledge gaps, we performed a comprehensive genomic, metabolomic, and bioactivity investigation of isolate VM-36. Long-read whole-genome sequencing generated a high-quality genome assembly, while comparative genomics, genome mining, and untargeted metabolomics revealed its biosynthetic potential and enabled the putative annotation of its major secondary metabolites. Among these, compound **1**, a decalin-containing tetramic acid (DTA), was identified as the major antibacterial metabolite exhibiting potent activity against both methicillin-sensitive and methicillin-resistant *Staphylococcus aureus*. Compared with previous studies on *Neocucurbitaria*, which were largely limited to fungal ecology or the characterization of diterpenoids with limited biological activities, this work expands the known metabolic diversity of the genus. Furthermore, the putative association of DTA-related metabolites with a candidate biosynthetic gene cluster provides a genomic framework for future investigation of their biosynthesis and genome-guided discovery of antibacterial natural products from this understudied fungal genus.

## 2 Materials and methods

### 2.1 Fungus isolation, morphology, and phylogenetic tree analysis

The fungus *Neocucurbitaria* sp. VM-36 was isolated from healthy leaves of *Vinca minor* in November, 2021 (Groningen, The Netherlands) as previously described (He et al., 2023). The strain was subsequently stored at 8 °C in the department of Chemical and Pharmaceutical Biology at the University of Groningen.

To investigate the morphology of this fungus, we inoculated it onto potato dextrose agar (PDA), malt extract agar (MEA), synthetically nutrient-poor agar (SNA), and Sabouraud dextrose agar (SDA) plates. After incubation at 25 °C for 28 days, as well as 77 days for SDA, we determined the colony characteristics, including color, diameter, and mycelium morphology. Fungal hyphae were stained with lactophenol blue dye, and their microscopic features were examined with an optical microscope (Olympus BX41). Digital images were captured using a Leica camera (Heerbrugg, Switzerland) connected to the microscope.

For the molecular identification of this fungus, we compared the sequence of its ITS region to 18 *Neocucurbitaria* strains, with *Fusarium oxysporum* Fo47 used as the outgroup, following the method described in our recent publication (He et al., 2023). We furthermore performed a multi-locus phylogenetic analysis using three barcode sequences: ITS, the RNA polymerase II subunits 2, and the large ribosomal subunit. Genome sequences of nine Cucurbitariaceae family strains, obtained from the Joint Genome Institute Genome Portal^1^ and National Center for Biotechnology Information^2^, were included alongside *F. oxysporum* Fo47 used as an outgroup. Sequences from the three loci were concatenated and aligned using ClustalW, and the Maximum Likelihood tree was generated in MEGA (v11) with 1,000 bootstrap replicates (Tamura et al., 2021).

### 2.2 Fermentation, extraction, and isolation

The fungus *Neocucurbitaria* sp. VM-36 was grown on PDA plates at 25 °C for 28 days. Five mycelial discs were used to inoculate a 3 L Erlenmeyer flask containing dextrose peptone yeast (DPY) medium. Seven independently inoculated flasks containing a total of 13.0 L medium were cultured in a shaker at 25 °C at a speed of 130 rpm. These independently inoculated flask cultures were maintained under identical fermentation conditions, and compound **1** was successfully obtained as the major metabolite from the fermentation system, indicating the reliability of the established culture conditions for metabolite production. After 30 days, the broth and mycelium were separated by filtration. The mycelium was ultrasonicated with EtOAc (three times, 0.52 L organic solvent each time, 1 h, 30 °C). The broth was extracted by EtOAc using liquid–liquid extraction (three times, 6.5 L solvent each time). The EtOAc extracts were combined and evaporated to dryness using a rotary evaporator (Hei-VAP Core Rotary Evaporators, Heidolph Instruments GmbH & CO. KG, Germany) to obtain a total of 1,404 mg crude extract. The crude extract was further dissolved in 80% acetonitrile-water solvent and centrifuged to obtain the supernatant, which was used for MPLC separation (BUCHI Reveleris™ X2-UV System) on a C_18_ column (FlashPure EcoFlex C_18_ 12 g column from Buchi®). The analytes were eluted with solution A (water) and solution B (acetonitrile) using a step-gradient program (10 min, 0% B; 5 min, 0–20% B; 10 min, 20% B; 5 min, 20–40% B; 10 min, 40% B; 5 min, 40–60% B; 10 min, 60% B; 5 min, 60–80% B; 10 min, 80% B; 5 min, 80–90% B; 10 min, 90% B) to give 7 fractions. The flow rate was 10.0 mL/min. UV detection was set at 210, 230, and 294 nm. Fractions F and G were separately concentrated by a rotary evaporator under reduced pressure at 35 °C, and further purified by a preparative liquid chromatography (Shimadzu LC-8A preparative liquid chromatograph system, SCL-10A vp system controller, SPD-10A UV–vis detector, SIL-10AP auto injector, and FRC-10A fraction collector) on a C_18_ column (VarioPrep high-performance liquid chromatography) Separation Column VP Nucleodur 100–5 C_18_ ec, (10 × 250 mm, 5 μm particle size) with 75% ACN isocratic elution. Column temperature was set at 40 °C. The flow rate was 7 mL/min, and UV detection was set at 290 nm. Fifteen fractions were obtained after separation. In order to obtain pure compounds, fractions 9, 10, and 11 were purified on a Shimadzu LC-10AT high-performance liquid chromatography system equipped with a Shimadzu SPD-M20A diode array detector and eluted on a C_18_ column (EC 100/4.6 NUCLEOSHELL RP 18, 4.6 × 100 mm, 2.7 μm particle size) with isocratic 75% ACN at 0.8 mL/min flow rate, to yield compound **1** (114 mg, retention time (t_R_) = 8.5 min). The relatively high recovery of compound **1** from the established fermentation system further demonstrates the feasibility of this culture protocol for preparative-scale production of this metabolite.

### 2.3 Structure characterization

In total 32 mg of compound **1** was dissolved in CDCl_3_ and used for NMR analysis. NMR spectra were recorded on a 400 MHz spectrometer (JEOL Ltd., Tokyo, Japan). Chemical shifts ( δ ) are reported in parts per million (ppm) and coupling constants (*J*) are reported in hertz (Hz). MestReNova 14 was used for data analysis.

Marfey’s method involving acid hydrolysis was used to determine the amino acid configuration in compound **1** according to the reported method (Gao et al., 2024) with minor modification. Compound **1** (0.2 mg) was dissolved in 2 mL of 6 M HCl and left at 110 °C for 16 h in a sealed tube. The hydrolysate was dried under a N_2_ flow. In an Eppendorf tube, 3.6 μmol of a 1% acetone solution of FDAA (N-(5-fluoro-2, 4-dinitrophenyl)-l-alaninamide) and 20 μmol of a 1 M solution of NaHCO_3_ were added to the hydrolysate, and 2.5 μmol of standards *D*-serine and *L*-serine, respectively. The reaction mixture was heated with frequent shaking over a hot plate at 40 °C for 1 h and then cooled to room temperature. Then, 20 μmol of 2 M HCl and 1 mL of MeOH were added to the reaction mixture. The samples were analyzed by low-resolution LC–MS, and molecular weights and retention times were compared with those of standard serines. Acetonitrile/water containing 0.1% formic acid was used as the mobile phase under a linear gradient elution mode at a 0.5 mL/min flow rate. A mass range of *m*/*z* 100–1,250 was covered with a scan time of 1 s, and data were collected in the positive ion mode. UV detection at 200–400 nm was performed by photodiode array detection.

UV and circular dichroism (CD) spectra of the compound **1** dissolved in ethanol were obtained using a JASCO V-660 spectrometer and JASCO J-815 CD spectrometer (JASCO corp., Tokyo, Japan), respectively. The wavelength scan was set to 210–400 nm. ECD calculations of two tautomers, (2*R*, 3*S*, 6*R*, 8*S*, 11*S*, 5’*S*)-A (C-1 keto/C-4′ enol) and (2*R*, 3*S*, 6*R*, 8*S*, 11*S*, 5’*S*)-B (C-4′ keto/C-1 enol), were performed following a reported reference (Zhang et al., 2016) with minor modifications as described below. Conformational sampling was carried out using RDKit^3^ (ETKDGv3), and resulting conformers were energy-minimized with the MMFF94 force field. Further geometry optimization and frequency analysis were performed at the B3LYP/6–31G(d) level with the polarizable continuum model solvent model for ethanol. Time-dependent density functional theory calculations were performed for excited-state properties. Conformers without imaginary frequencies were subjected to ECD calculations at the B3LYP/6–31 + G(d) without applying a solvent model due to the high computational cost and frequent convergence difficulties associated with the inclusion of solvent effects. For each tautomer, the six conformers with the lowest electronic energies at the TD-DFT level were selected. The ECD spectra of these conformers were simulated using SpecDis over the wavelength range of 210–400 nm, with a half-bandwidth of 0.16 and 0.25 eV, and were subsequently Boltzmann-weighted according to their relative populations. All quantum chemical calculations were performed using Gaussian 16. The experimental and calculated ECD spectra were compared to facilitate the assignment of the absolute configuration.

### 2.4 Bacteria and antibacterial susceptibility assay

Several bacterial strains were used in this assay (Supplementary Table S1), including the following strains from the American Type Culture Collection (ATCC) and the National Collection of Type Cultures (NCTC) of Public Health England: *S. aureus* ATCC 29213 (MSSA), *S. aureus* NCTC 8325 (MSSA), *Enterococcus faecalis* ATCC 29212, *Enterobacter cloacae* ATCC 13047, *Klebsiella pneumoniae* ATCC 13883, *Acinetobacter baumannii* ATCC 17978, *Pseudomonas aeruginosa* ATCC 27853, and *Escherichia coli* ATCC 25922. In addition, we used *S. aureus* strain Newman (Lorenz and Duthie, 1952), *S. aureus* NE1688 (Sle1) (MRSA) from the Nebraska transposon library (Fey et al., 2013), which carries a transposon insertion in the chromosomal *sle1* gene (Wang M. et al., 2024), and the two clinical MRSA isolates D15 and D17, which were, respectively, associated with community- and hospital-acquired infections (Mekonnen et al., 2017). The minimum inhibitory concentrations (MICs) and minimum bactericidal concentrations (MBCs) of compound **1** were determined using the broth microdilution method in 96-well microtiter plates according to the guidelines from the European Committee for Antibacterial Susceptibility Testing (The European Committee on Antimicrobial Susceptibility Testing [EUCAST], 2022). Detailed procedures are described in the Supplementary Information Methods. The lowest concentration of compound **1** that completely inhibited bacterial growth was determined as the MIC, and the MBC was determined as the lowest concentration of compound **1** that reduced the viable bacterial count on agar plates by 99.9%. Vancomycin and daptomycin were used as positive controls. Bacteria treated with the respective vehicles were used as negative control. All tests were performed in triplicates.

### 2.5 Determination of bacterial growth kinetics and drug interaction assays

Bacterial growth kinetics of compound **1**, vancomycin, and daptomycin against 1 MSSA and 4 MRSA strains at final concentrations of 2, 4, and 8 μg/mL were monitored by measuring the optical density at 600 nm (OD_600_) values. Bacterial viability before compound addition and at the endpoint was determined by colony-forming unit (CFU) enumeration, as detailed in the Supplementary Information Methods. All experiments were performed in triplicate.

The drug interaction of compound **1** (**A**) with daptomycin (**B**) or vancomycin (**C**) against the three MRSA strains, *S. aureus* USA300, D15 and D17 was determined using the checkerboard assay in 96 well microplates. In total, 35 combinations were tested, which included five concentrations of compound **1** (final concentrations 2–0.125 μg/mL) together with seven concentrations of daptomycin or vancomycin (2–0.03125 μg/mL). All treatments were tested in 3 independent experiments (*n* = 3). The assay was performed at a final concentration of 5 × 10^4^ CFUs/ well for each strain. After 24 h of incubation at 36 ± 1 °C, MIC values of drug A, drug B, drug C, and their combinations were determined. The fractional inhibitory concentration index (FICI) was calculated based on previously reported methods (Berenbaum, 1977; Betts et al., 2016), and the equations were listed as follows:


$$\text{FICA}=\frac{\mathrm{MIC}\;\text{of}\;A\;\text{in}\;a\;\text{combination with}\;B}{\mathrm{MIC}\;\text{of compound}\;A}$$

$$\text{FICB}=\frac{\mathrm{MIC}\;\text{of}\;B\;\text{in}\;a\;\text{combination with}\;A}{\mathrm{MIC}\;\text{of compound}\;B}$$

FICI=FICA+FICB

The same calculation was used for the combinations of **A** and **C**. These values were analyzed by nonparametric models based on the Loewe additivity model (Russ and Kishony, 2018). FICI results were interpreted as synergistic (FICI ≤ 0.5), additive (0.5 < FICI ≤ 1), indifferent (1 < FICI ≤ 4) or antagonistic (FICI > 4) (Odds, 2003; Mathers, 2015). The lowest values of FICI (FICI_Min_), the median FICI (FICI_Med_), and the highest FICI (FICI_Max_) were calculated for each combination and in each strain. Outcomes of these experiments were plotted in heatmaps using GraphPad Prism version 8.0.1.

### 2.6 Hemolytic activity and mammalian-cell cytotoxicity assays

To evaluate the effect of compound **1** on human erythrocytes, a hemolysis assay was performed as detailed in the Supplementary Information Methods. Briefly, compound **1** was dissolved in DMSO at concentrations of 200, 100, 10, and 1 μg/mL. DMSO at 1%, 0.5%, 0.05 and 0.005% v/v concentrations were used as vehicle control. Sodium dodecyl sulfate at a final concentration of 0.1% w/v was used as the positive control and Dulbecco’s phosphate-buffered saline pH 7.2 served as a negative control. Vancomycin and daptomycin were included at the same final concentrations as compound **1**. Double-distilled water and NaCl 0.9% w/v were evaluated as their blanks, respectively. All groups were tested in triplicates. Blood donations from healthy volunteers were collected based on written informed consent with approval of the medical ethics committee of the University Medical Center Groningen (UMCG; approval no. Metc2012-375), and in accordance with the Declaration of Helsinki guidelines.

The mammalian-cell cytotoxicity of compound **1** was further evaluated in immortalized human bronchial epithelial 16HBE14o- cells using a resazurin-based assay, as described in detail in the Supplementary Information. Briefly, 16HBE14o- cells were seeded at 4000 cells/well in 96-well plates and incubated for 3 days before treatment with compound **1** at 1, 10, 100, and 200 μg/mL. Corresponding DMSO concentrations were included as vehicle controls, while vancomycin and daptomycin were tested at the same concentrations for comparison. SDS (0.1%, w/v) and supplemented eMEM medium were used as positive and negative controls, respectively. Following 24 h of treatment, cell metabolic activity was determined using a resazurin-based assay by measuring fluorescence. The percentage of cell metabolic activity was calculated relative to the negative control, and the IC_50_ value was estimated by linear regression analysis. Each treatment was tested in three technical replicates in two independent experiments.

### 2.7 Genome mining

#### 2.7.1 DNA extraction, whole-genome sequencing and assembly

DNA extraction, library preparation, sequencing, and assembly were conducted as described recently (He et al., 2023). The raw reads were base-called using Guppy version 6.1.5 (Oxford Nanopore Technologies, Oxford, UK) in GPU mode using the dna_r10. 4_ e8.1_ sup. Cfg model (Wick et al., 2019). The base-called reads were subsequently filtered to a minimum length of 2 kb and a minimum quality of Q10 using NanoFilt (version 2.8.0) (De Coster et al., 2018). NanoPlot (version 1.40.0) (De Coster et al., 2018) was used to evaluate the filtered reads. Assembly was performed using Flye (version 2.9-b1778). The quality of the genome assembly was evaluated using QUAST v5.1.0rc1 (Gurevich et al., 2013). Bandage (version 0.8.1)(Ryan R. Wick et al., 2015) was used to visualize the newly assembled genome of *Neocucurbitaria* sp. VM-36. The draft assembly was subsequently polished in two rounds: first using Racon version 1.4.10 with default settings (Vaser et al., 2017) and then Medaka version 0.11.5 with default settings. The completeness of the assembly was evaluated using BUSCO 5.4.3 (ascomycota_odb10 dataset).

#### 2.7.2 Gene prediction and functional annotation

Genome annotation was carried out using the online platform Genome Sequence Annotation Server^4^, which provides a pipeline for whole-genome structural and functional annotation (Jodi L. Humann et al., 2019). The sequencing data and genome assembly for this study were deposited in the European Nucleotide Archive at EMBL-EBI under accession number PRJEB97715. Gene Ontology annotation (Törönen and Holm, 2022), carbohydrate-active enzymes annotation (Zhang et al., 2018), tRNA (Chan and Lowe, 2019) and rRNA prediction^5^ were further performed. The biosynthetic potential of *Neocucurbitaria* sp. VM-36, along with that of six other strains with available whole genome sequences, including *Cucurbitaria berberidis* CBS 394.84, *Neocucurbitaria cava* IMI 356814, *Parafenestella ontariensis* EI-6, *Pyrenochaeta* sp. DS3sAY3a, *Pyrenochaeta inflorescentiae* CORFU0001, and *Fenestella fenestrata* ATCC 66461, was analyzed by antiSMASH fungal version 7.0.1 (Blin et al., 2023) under relaxed detection strictness and default settings.

### 2.8 Untargeted metabolomics

#### 2.8.1 Extraction of fungal secondary metabolites

Fungal mycelium of *Neocucurbitaria* sp. VM-36 was separately transferred to two different culture media, including PDA solid medium ( Φ 35 mm × 10 mm plate) and DPY liquid medium (20 mL medium in Erlenmeyer flask). Solid plates were incubated at 25 °C for 28 days, and their SMs were extracted with organic solvent as recently described (He et al., 2023). Fungal mycelium in DPY liquid medium was cultured at 25 °C for 28 days, with shaking at 130 rpm. The fungal mycelium and broth were mixed with 20 mL EtOAc, sonicated and rotated in a rotating mixer for 1 h, respectively. The organic phase was subsequently collected and dried under a gentle stream of N_2_. The dried extract was resuspended in 1 mL of 1:1 MeOH-MilliQ water (*v*/*v*) and filtered with 0.45 μm polytetrafluoroethylene filters (Screening Devices BV company, The Netherlands), and then subjected to liquid chromatography-coupled tandem mass spectrometry analysis.

#### 2.8.2 Molecular networking-based liquid chromatography-coupled tandem mass spectrometry analysis

Liquid chromatography-coupled tandem mass spectrometry analysis of crude EtOAc extracts was performed as described before (He et al., 2023). Fresh PDA and DPY media were used as blank control groups. The acquired data was processed by Thermo Scientific FreeStyle software version 1.8. Untargeted metabolomic analysis was conducted using the online workflow on the Global Natural Products Social Molecular Networking (GNPS) website^6^ according to our previously described method (Wang et al., 2016; He et al., 2023). The raw mass spectrometry data were deposited on GNPS under the accession number MassIVE ID: MSV000098902. A molecular networking analysis^7^ was conducted using the default settings in GNPS, except that the precursor ion mass tolerance and the MS/MS fragment ion tolerance were both set to 0.02 Da. The results were visualized in Cytoscape version 3.9.1 (Otasek et al., 2019). Nodes from the blank PDA and DPY media were used as background and omitted to obtain the final molecular network.

## 3 Results

### 3.1 Isolate VM-36 from *Vinca minor* is a *Neocucurbitaria* species

During our bioprospecting of fungal endophytes from the traditional medicinal plant *Vinca minor*, strain VM-36 emerged as a particularly promising isolate. Initial bioactivity screening via disk diffusion assays revealed that the crude ethyl acetate extract of VM-36 exerted robust inhibitory effects against the Gram-positive indicator bacterium *B. subtilis* 168 (Supplementary Figure S1). Preliminary taxonomic assignment using ITS sequencing placed this strain within *Neocucurbitaria*, a recently established and relatively under-investigated genus in the family Cucurbitariaceae.

Since the genomic architecture and biosynthetic landscape of the *Neocucurbitaria* genus remain largely unexplored, we initiated a comprehensive multi-omics characterization of VM-36. We performed high-depth, long-read sequencing of its genomic DNA using Oxford Nanopore Technology (ONT), yielding 870,296 raw reads (5.5 Gbp) with an N50 of 5.6 kb (Table 1). The *de novo* assembly resulted in a 32.8 Mbp genome distributed across 19 contigs (Supplementary Figure S2). Assessment via BUSCO confirmed a completeness of 97.2%, providing a highly contiguous and reliable reference for further analysis. Structural annotation of this high-quality assembly predicted 11,817 protein-coding genes, 29 rRNAs, and 120 tRNAs.

**Table 1:** Reads and assembly statistics of whole-genome sequencing of *Neocucurbitaria* sp. VM-36.

Metric	Unfiltered	Filtered (>2 kb)
No. of bases (Gb)	5.5	5.2
No. of reads	870,296	590,512
N50 (kb)	5.5	7.3
Mean read length (bp)	6,422.9	9,074.5
Mean read quality	16.1	17.1
Coverage	130×	
Polishing steps	Medaka	
No. of contigs (coverage ≥ 40×)	19	
Genome size (Mb)	32.8	
GC (%)	50	
BUSCO (Ascomycota_odb10) (%)	97.2	
Number of the protein-coding genes	11,817	
tRNA genes	120	
rRNA genes	29	
Proteins with a predicted Pfam domain	9,806	
Carbohydrate-active enzymes	570	

Next, we performed phylogenetic analyses to more accurately locate our isolate within the *Neocucurbitaria* genus. Both single-locus (ITS) and multi-locus (RNA polymerase II subunits 2, ITS, and large ribosomal subunit) analyses consistently showed that *Neocucurbitaria* sp. VM-36 is closely related to *Neocucurbitaria salicis-albae* CBS 144611 (Figure 1), a species previously isolated from a *Salix alba* twig in Germany (Crous et al., 2019).

**Figure 1:**
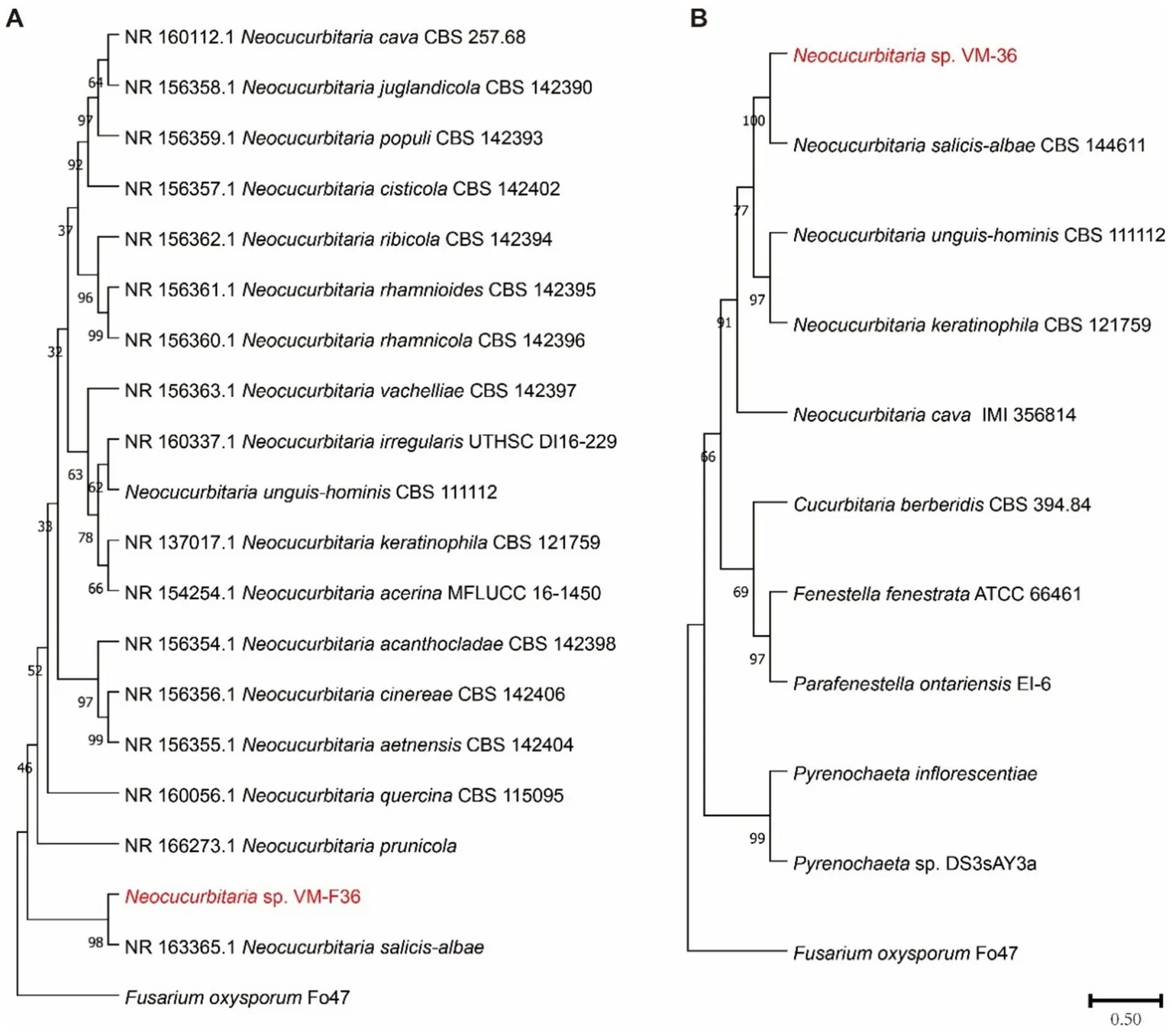
Phylogenetic analysis of *Neocucurbitaria* sp. VM-36. **(A)** Maximum Likelihood phylogram of *Neocucurbitaria* sp. VM-36 based on the ITS sequence. **(B)** Maximum Likelihood phylogenetic tree based on concatenated nucleotide sequences of RNA polymerase II subunits 2, ITS, and large ribosomal subunit. *F. oxysporum* Fo47 was used as an outgroup in both analyses.

The morphological features of *Neocucurbitaria* sp. VM-36 were evaluated in four nutrient media (Figure 2). Mycelial growth and conidiation were notably enhanced on PDA and MEA compared to SNA and SDA. On PDA, colonies reached 27–37 mm in diameter after 28 days, characterized by black, textured mycelia. MEA supported the most rapid growth within 28 days (35–40 mm), producing brown-and-white floccose aerial hyphae. Conversely, growth was restricted on SNA (15–25 mm, white velvety) and SDA (12–20 mm, yellow and granular). Interestingly, prolonged incubation on SDA until day 77 allowed colonies to reach 28–37 mm, developing a brown textured surface with a black peripheral reverse. Microscopic observation (Figure 2B) identified spherical pycnidia containing conidiophores and asexual conidiospores. Furthermore, thick-walled chlamydospores were observed exclusively on SDA, suggesting a specialized adaptation for survival under nutrient-limiting conditions.

**Figure 2:**
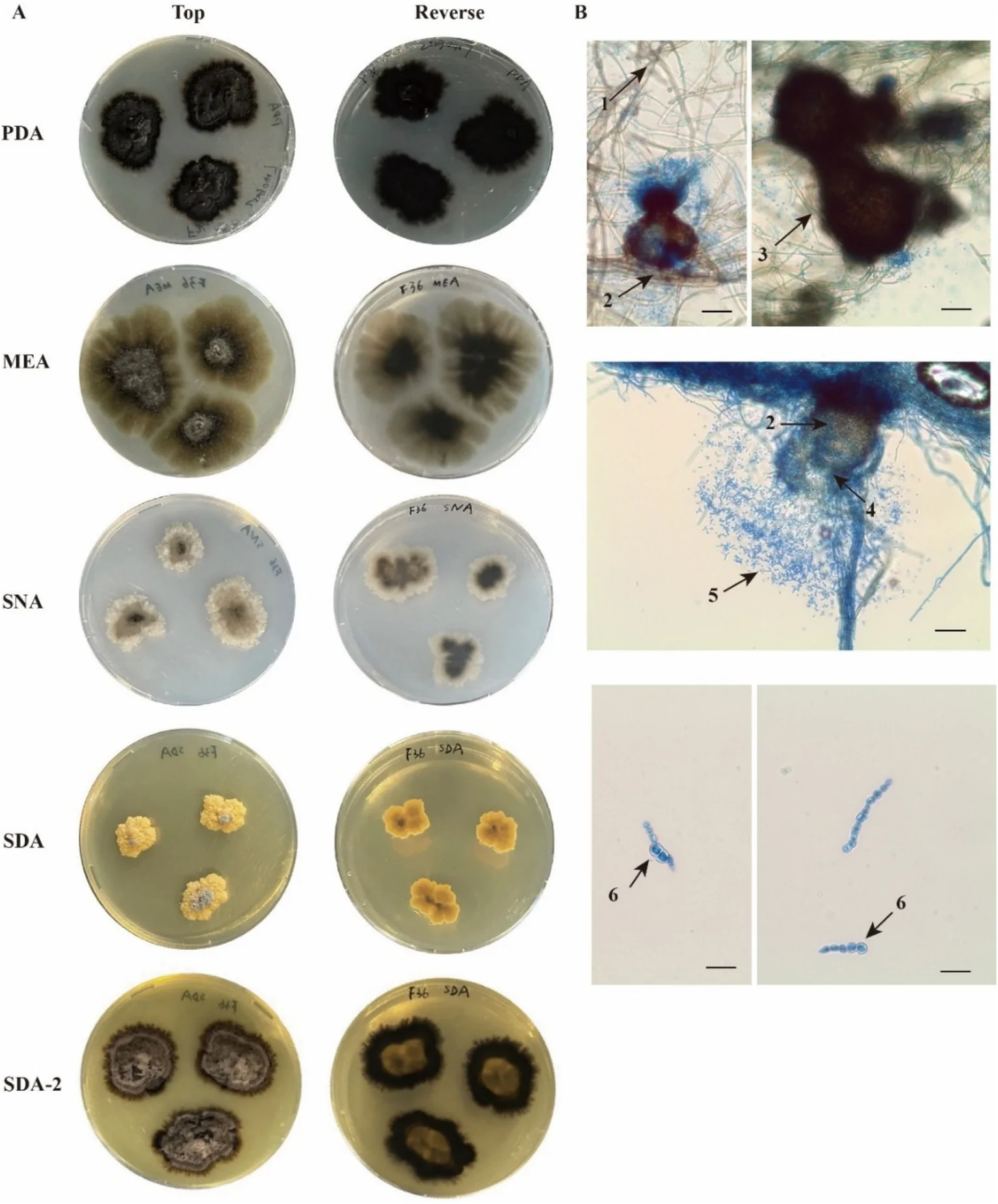
Macroscopic **(A)** and microscopic **(B)** characteristics of *Neocucurbitaria* sp. VM-36 on different culture media (PDA, MEA, SNA and SDA), incubated at 25 °C for 28 days or 77 days (SDA-2). (1) Hyphae; (2) Pycnidium- Conidiomata; (3) Pycnidia; (4) Conidiophores; (5) Conidia; and (6) Chlamydospore chains formed on SDA medium. Scale bars = 50 μm.

### 3.2 Isolation and structural characterization of compound **1**

We analyzed the EtOAc extract of *Neocucurbitaria* sp. VM-36 by LC–MS and observed one dominant peak at retention time (t_R_) 20.33 min next to several minor constituents (Supplementary Figure S3). We purified the major compound (compound **1**) from large-scale fungal culture as a white amorphous powder. Its molecular formula was established to be C_23_H_33_NO_4_ based on the observed *m/z* 388.2478 ([M + H]^+^ ion, calculated 388.2493). Based on MS2 fragmentation, compound **1**, belongs to the decalin-containing tetramic acids (DTAs) with 8 degrees of unsaturation and serine giving rise to the tetramic acid moiety (vide infra). The UV absorbance spectrum of compound **1** in ethanol with maxima at λ _max_ ( ε ) 252 (4.05) and 291.8 (4.08) nm (Supplementary Figure S4A) is similar to the structurally related compound phomasetin (Nogawa et al., 2018). The acid hydrolysate of compound **1** was derivatized with FDAA, and the resulting FDAA-Ser derivative was compared with those obtained from authentic *L*-serine and *D*-serine standards. The retention time of the FDAA-Ser signal from the sample was consistent with that of the *L*-serine derivative (Supplementary Figure S4B), suggesting the *L*-configuration for the serine-derived moiety in compound **1**, consistent with that reported for phomasetin (Nogawa et al., 2018).

Next, we determined the structure of compound **1** by NMR. According to the 1D NMR (Table 2 and Supplementary Figures S5, S6) and MS2 data (Supplementary Table S2), compound **1** has one -C_2_H_2_ unit less and two sp^2^-carbon signals less than phomasetin, indicating the absence of one double bond in compound **1** (Mao et al., 2020; Singh et al., 1998). This feature matches that of CJ-21058 (Sugie et al., 2002) and the stereoisomers, hyalodendrin A and B (Mao et al., 2020). The 2D NMR spectra of compound **1**, including ^1^H-^1^H COSY, ^1^H-^13^C HSQC, ^1^H-^13^C HMBC, TOCSY, and NOESY, are provided in Supplementary Figures S7–S11, with key correlations labeled. The ^1^H-^1^H COSY and ^1^H-^13^C HMBC correlations used for the structural assignment are shown in Figure 3A. As shown in Table 2, the NMR data of compound **1** are comparable to those of CJ-21058 in MeCN-*d*_3_ and hyalodendrins A and B in CDCl_3_ (Sugie et al., 2002; Mao et al., 2020), all of which contain a decalin ring system and a tetramic acid moiety. On the basis of the HMBC correlations of H-12/C-1, H-5’/C-4’, and H-6’/C-4’, the ^13^C signals at δC 199.31 and 190.66 ppm are assigned to C-1 and C-4’, respectively. The relative chemical shifts of these carbons are consistent with a keto-enol distribution in which C-1 exhibits a more carbonyl-like character and C-4’ a more enolized character. Given the known keto-enol tautomerism of tetramic acid derivatives, however, the exact distribution of the tautomeric forms could not be unambiguously established from the available NMR data. The structure of compound **1** is therefore represented by the C-1 keto/C-4’ enol tautomeric form for clarity, while the contribution of alternative tautomeric forms cannot be excluded. The ^1^H-^1^H NOESY spectrum (Figure 3B) of compound **1** exhibits key correlations for H-3/H-16, H-3/H-12, H-6/H-12, H-17/H-8, H-6’eq/H-5’, and H-11/H-16. Additional correlations of H-6/H-7eq, H-6/H-8, H-8/H-7eq, H-6/H-9eq and H-9eq/H-10ax allow for the assignment of the stereochemical framework.

**Table 2:** ^1^H NMR (400 MHz) and ^13^C NMR (100 MHz) data of compounds **1** (in CDCl_3_), compared with the previously reported compounds CJ-21058 (in MeCN-*d*_3_) and hyalodendrins A and B (in CDCl_3_).

Position	Compound 1		CJ-21058 (Sugie et al., 2002)		Hyalodendrin A (Mao et al., 2020)		Hyalodendrin B (Mao et al., 2020)	
	$\delta_{H},$ Multi. (*J* in Hz)	$\delta_{C},$ Type	$\delta_{H},$ Multi. (*J* in Hz)	$\delta_{C},$ Type	$\delta_{H},$ Multi. (*J* in Hz)	$\delta_{C},$ Type	$\delta_{H},$ Multi. (*J* in Hz)	$\delta_{C},$ Type
1	–	199.31, C	–	197.7, C	–	199,0, C	–	199.2, C
2	–	49.46, C	–	49.8, C	–	49.4, C	–	49.5, C
3	3.05, m	49.37, CH	3.04, br	50.3, CH	3.06, m	49.4, CH	3.07, m	49.3, CH
4	–	131.99, C	–	132.9, C	–	132.0, C	–	132.0, C
5	5.16, s	125.69, CH	5.15, brs	126.6, CH	5.15, s	125.6, CH	5.16, s	125.6, CH
6	1.76, m	39.11, CH	1.81, m	40.0, CH	1.82, m	39.0, CH	1.82, m	39.2, CH
7	0.88, m (eq) 1.79, m (ax)	42.50, CH_2_	0.83, dd (12.0, 11.9) 1.76, m	43.2, CH_2_	0.87, m 1.78, m	42.6, CH_2_	0.87, m 1.78, m	42.5, CH_2_
8	1.49, m	33.53, CH	1.47, m	34.3, CH	1.50, m	33.5, CH	1.50, m	33.5, CH
9	1.10, m (eq) 1.77, m (ax)	35.77, CH_2_	1.03, m 1.70, m	36.6, CH_2_	1.11, m 1.75, m	35.8, CH_2_	1.11, m 1.75, m	35.8, CH_2_
10	1.03, m (eq) 1.96, br. d (11.9) (ax)	28.20, CH_2_	1.03, m 1.93, br	28.9, CH_2_	1.02, m 1.95, br. d (11.2)	28.3, CH_2_	1.02, m 1.96, br. d (11.9)	28.2, CH_2_
11	1.64, m	39.63, CH	1.63, m	40.5, CH	1.66, m	39.7, CH	1.67, m	39.7, CH
12	1.39, s	13.63, CH_3_	1.33, brs	14.1, CH_3_	1.42, s	13.8, CH_3_	1.40, s	13.7, CH_3_
13	5.16, s	130.64, CH	5.10, m	131.5, CH	5.11, m	130.6, CH	5.12, m	130.6, CH
14	5.23, m	127.60, CH	5.19, m	128.5, CH	5.23, dq (15.4, 6.0)	127.7, CH	5.24, m	127.5, CH
15	1.55, d (6.2)	17.87, CH_3_	1.48, brd (5.4)	17.9, CH_3_	1.53, d(6.0)	22.2, CH_3_	1.55, d (5.7)	17.9, CH_3_
16	1.59, s	22.31, CH_3_	1.53, brs	22.4, CH_3_	1.60, s	22.2, CH_3_	1.59, s	22.2, CH_3_
17	0.91, d (6.5)	22.55, CH_3_	0.87, d (6.6)	22.7, CH_3_	0.91, d (6.7)	22.5, CH_3_	0.91, d (6.4)	22.5, CH_3_
2’	–	177.28, C	–	178.1, C	–	177.1, C	–	177.2, C
3’	–	100.02, C	–	101.6, C	–	100.2, C	–	99.9, C
4’	–	190.66, C	–	191.4, C	–	190.7, C	–	190.5, C
5’	3.62, dd (4.9, 4.0)	66.60, CH	3.54, t (2.9)	68.7, CH	3.65, br. t (4.4)	66.2, CH	3.62, t (4.4)	66.7, CH
6’	3.88, m (ax) 4.02, dd (11.5, 3.9) (eq)	60.05, CH_2_	3.84, dd (12.0, 2.8) 3.76, dd (12.0, 3.0)	59.7, CH_2_	4.06, br. dd (11.5, 3.7) 3.85, dd (11.3, 5.0)	60.2, CH_2_	4.01, br. dd (11.5, 3.7) 3.88, dd (11.0, 4.0)	60.5, CH_2_
7’	3.05, s	27.34, CH_3_	2.94, s	27.4, CH_3_	3.04, s	27.2, CH_3_	3.05, s	27.3, CH_3_

**Figure 3:**
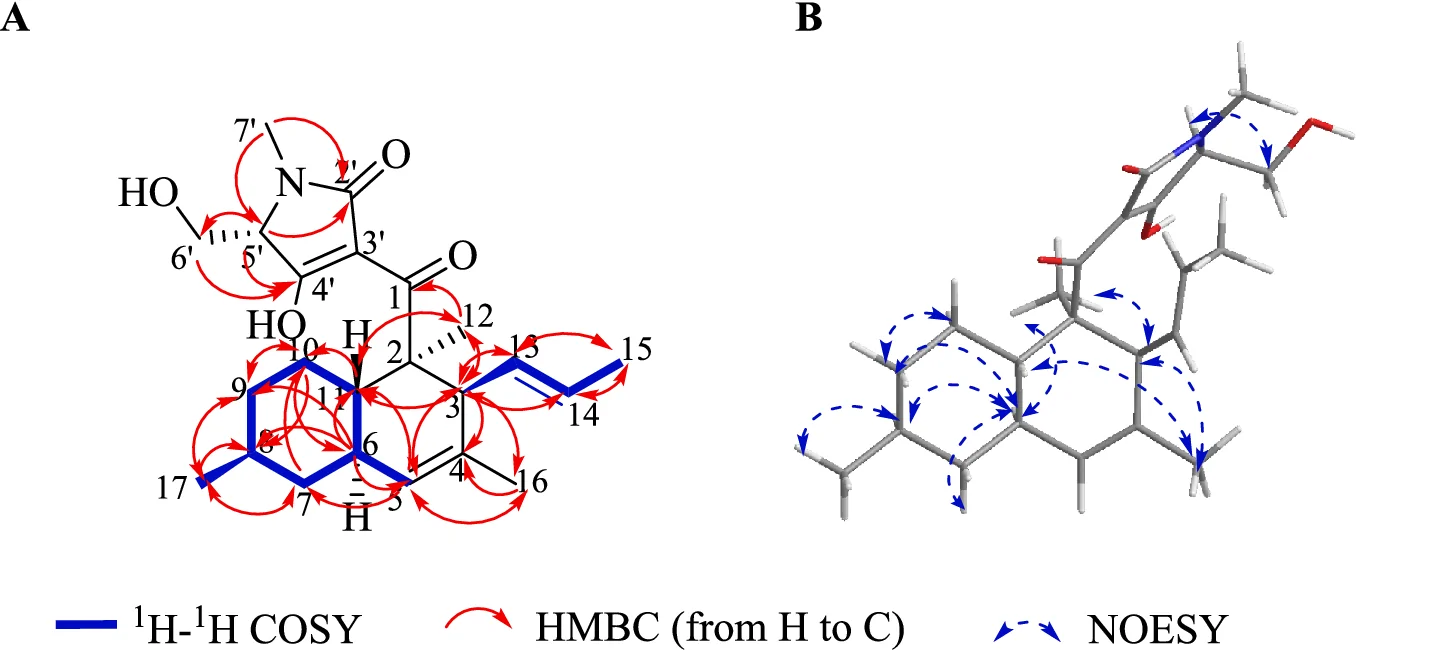
Physico-chemical characterization of compound **1**. Chemical structure with key two-dimensional NMR correlations (COSY and HMBC **(A)** and NOESY **(B)**).

The absolute configuration of compound **1** is tentatively assigned as (2*R*, 3*S*, 6*R*, 8*S*, 11*S*, 5’*S*) by comparing the experimental ECD spectrum with the calculated data of both tautomeric forms (C-1 keto/C-4’ enol, A; C-4’ keto/C-1 enol, B) (Supplementary Figures S4C, S13 and Supplementary Table S3). Although the calculated Δε values do not fully match the experimental intensities, as commonly encountered in TD-DFT calculations, these findings support, though do not conclusively establish, the proposed structural assignment of compound **1**. Taken together with NMR data, these results suggest that compound **1** may exist as a mixture of the two tautomeric forms, (2*R*, 3*S*, 6*R*, 8*S*, 11*S*, 5’*S*)-A and (2*R*, 3*S*, 6*R*, 8*S*, 11*S*, 5’*S*)-B, with the latter corresponding to hyalodendrin A.

### 3.3 Compound **1** exhibits potent antibacterial activity against multiple MSSA and MRSA strains

As the crude extract of *Neocucurbitaria* sp. VM-36 exerted a strong antibacterial effect against *B. subtilis* 168 (Supplementary Figure S1), we assessed the possible antimicrobial activity of compound **1** against a representative panel of ESKAPE(E) pathogens. The results show that it exerts strong inhibitory effects against several Gram-positive bacterial strains, including *E. faecalis* ATCC 29212 (MIC: 4 μg/mL), *S. aureus* ATCC 29213, *S. aureus* NCTC 8325, *S. aureus* Newman (MIC: 1 μg/mL), and *S. aureus* HG001 (MIC: 2 μg/mL). In contrast, compound **1** was inactive against the Gram-negative bacterial strains *K. pneumoniae* ATCC 13883, *A. baumannii* ATCC 17978, *P. aeruginosa* ATCC 27853, *E. cloacae* ATCC 13047 and *E. coli* ATCC 25922 (MICs: > 64 μg/mL) (Supplementary Table S4).

We further determined the MIC and MBC values of compound **1** against four MRSA strains, including the *S. aureus* USA300 wild-type strain, two clinical isolates of *S. aureus* USA300 (D15 and D17), and various transposon mutant *S. aureus* USA300 derivatives (Fey et al., 2013), as well as the MSSA reference strain *S. aureus* ATCC 29213 (Table 3). The vancomycin and daptomycin breakpoints for these strains are shown in Supplementary Table S5. Compound **1** shows strong antibacterial activity against both MRSA and MSSA strains (MIC: 0.5–1 μg/mL), similar to the positive controls vancomycin (MIC: 1 μg/mL) and daptomycin (MIC: 0.5–2 μg/mL). The MBC values of compound **1** (MBC: 2–4 μg/mL) were slightly higher than those of vancomycin and daptomycin (MBC:1–2 μg/mL). With MBC/ MIC ratios ≤ 4, we conclude that compound **1** shows bactericidal effects on *S. aureus* USA300, D15 and D17, and ATCC 29213. Strain *S. aureus* NE1688 (Sle1) (Fey et al., 2013) appears to be more sensitive to growth inhibition by low concentrations of compound **1**, but it is similarly sensitive to lethal doses as the other strains.

**Table 3:** Antibacterial activity evaluation of compound **1** against *S. aureus*.

Strain	Compound 1			Vancomycin		Daptomycin	
	MIC (μg/mL)	MBC (μg/mL)	MBC/ MIC ratio	MIC (μg/mL)	MBC (μg/mL)	MIC (μg/mL)	MBC (μg/mL)
*S. aureus* ATCC 29213 (MSSA)	1	2	2	1	1	0.5	1
*S. aureus* USA300 (MRSA)	1	4	4	1	1	1	1
*S. aureus* clinical isolate D15 (MRSA)	1	2	2	1	2	1	2
*S. aureus* clinical isolate D17 (MRSA)	1	4	4	1	1	1	1
*S. aureus* USA 300 NE1688 (Sle1) (MRSA)	0.5	4	8	1	2	2	2

### 3.4 Antibacterial kinetics, combinatorial effects, hemolytic activity, and mammalian-cell cytotoxicity of compound **1**

We also analyzed the bacterial growth curves of different *S. aureus* strains in the presence of compound **1** at different concentrations higher or equal than the MBC (2, 4, and 8 μg/mL) to monitor the dose-dependent change of optical density (Figure 4). After the exposure of bacterial cultures in mid-exponential phase to compound **1**, the bacterial growth was inhibited as noted by the decline in the OD_600_ values. Also, the maximum OD_600_ values of all bacterial cultures were significantly reduced in the presence of compound **1** compared to the vehicle controls (DMSO). This suggests that compound **1** not only has a dose-dependent bactericidal activity but also that it possibly induces bacterial lysis, particularly for the MRSA isolates D15, D17 and NE1688, with the strongest effects observed at 8 μg/mL. To further corroborate this observation, we assessed the bacterial viability before and after 40 h treatment by counting CFUs per mL liquid culture (Supplementary Figure S14). Compared with the positive controls daptomycin and vancomycin, compound **1** exerted stronger bactericidal activity against *S. aureus* ATCC 29213, USA300, D15 and D17 at 8 μg/mL and 4 μg/mL, with no bacterial colonies observed or a decrease up to 3 orders of magnitude (from ~ ×10^10^ CFUs/mL to ~ ×10^7^ CFUs/mL). Against *S. aureus* NE1688 (Sle1), compound **1** showed greater bactericidal activity than both controls at 8 μg/mL, but was less effective than daptomycin at 4 μg/mL. As expected, at the lowest tested concentration (2 μg/mL), none of the compounds exerted a significant bactericidal effect.

**Figure 4:**
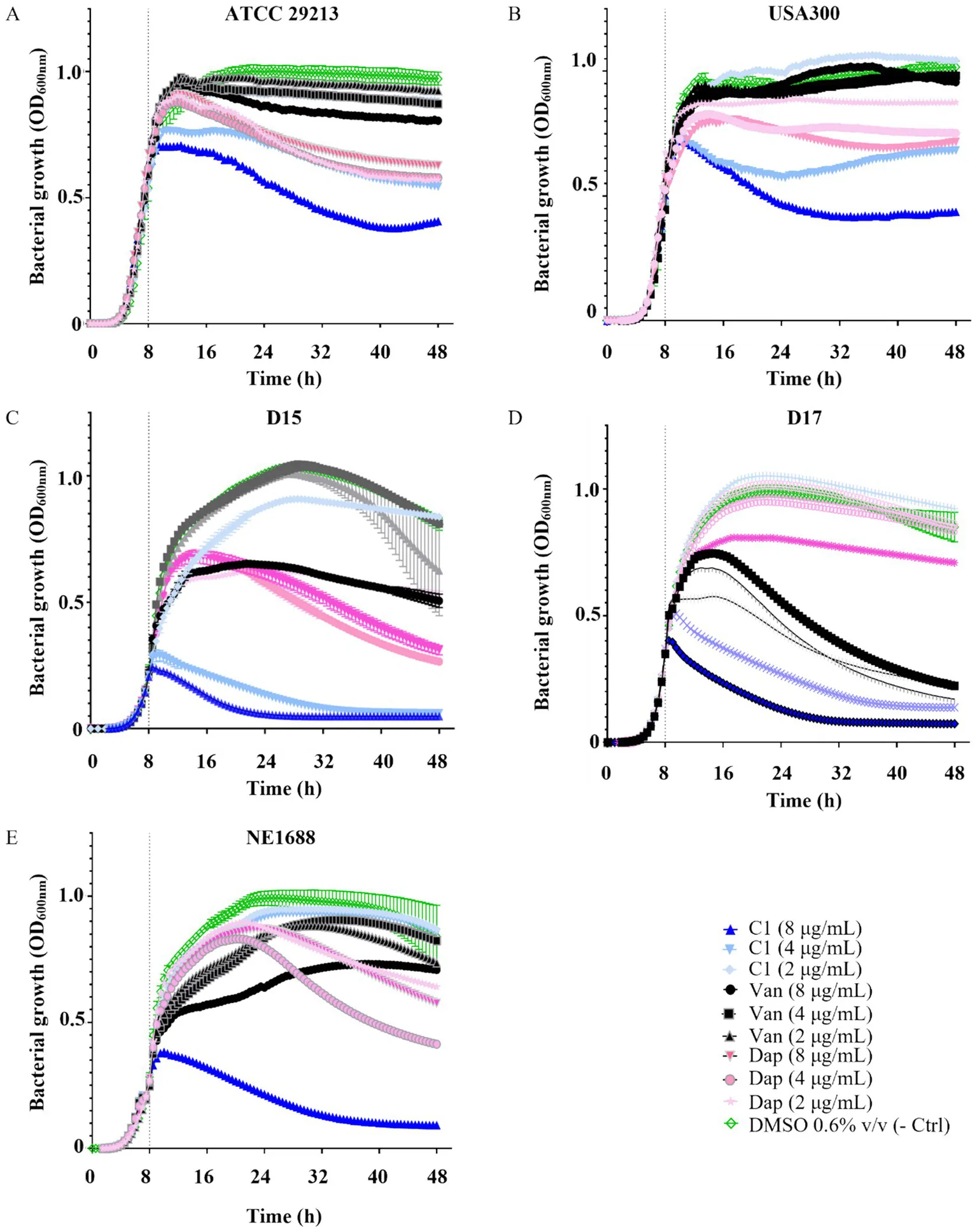
Growth curves of 5 *S. aureus* strains in the presence of compound **1**, or the positive controls vancomycin and daptomycin at 2, 4 and 8 μg/mL. **(A)** *S. aureus* ATCC 29213 (MSSA); **(B)** *S. aureus* USA300 (MRSA); **(C)** *S. aureus* D15 clinical isolate (MRSA); **(D)** *S. aureus* D17 clinical isolate (MRSA); and **(E)** *S. aureus* NE1688 (MRSA). The scattered line (---) in the *x*-axis indicates the time point at which the compound was added (*t* = 8 h). Results are expressed as mean OD_600nm_ values and error bars represent standard deviation (*n* = 3).

Next, we assessed the combined effects of compound **1** with daptomycin or vancomycin against a MSSA strain (*S. aureus* ATCC 29213) and two MRSA strains (*S. aureus* D15, and D17) (Figure 5). Among the 35 treatment combinations evaluated in the checkerboard assay, no antagonistic effects were observed. Most interactions were classified as indifferent, with a minimum fractional inhibitory concentration index (FICI_min_) of 1.25. Notably, the combination of compound **1** and daptomycin exhibited an additive effect against the clinical isolate D17 (FICI_min_ = 0.625). The additive effect of compound **1** with daptomycin observed for D17 MRSA, in contrast to the other MRSA strain D15 and the *sle1* mutant, could possibly be explained by different, as of yet undefined biological features. For instance, a higher cell-wall permeability or a different cell-wall composition in D17 compared to the other strains may increase its sensitivity to the daptomycin-compound **1** combination treatment (Wang M. et al., 2024). Or different drug resistance-related genetic elements could be present in the strains, making D17 less resistant to the treatment (Mekonnen et al., 2017). These findings suggest that compound **1** is compatible with established antibiotics. The absence of antagonism, particularly in combination with daptomycin, indicates its potential to complement the antibacterial profile of existing treatments against specific MRSA strains, such as D17, without diminishing antibiotic potency.

**Figure 5:**
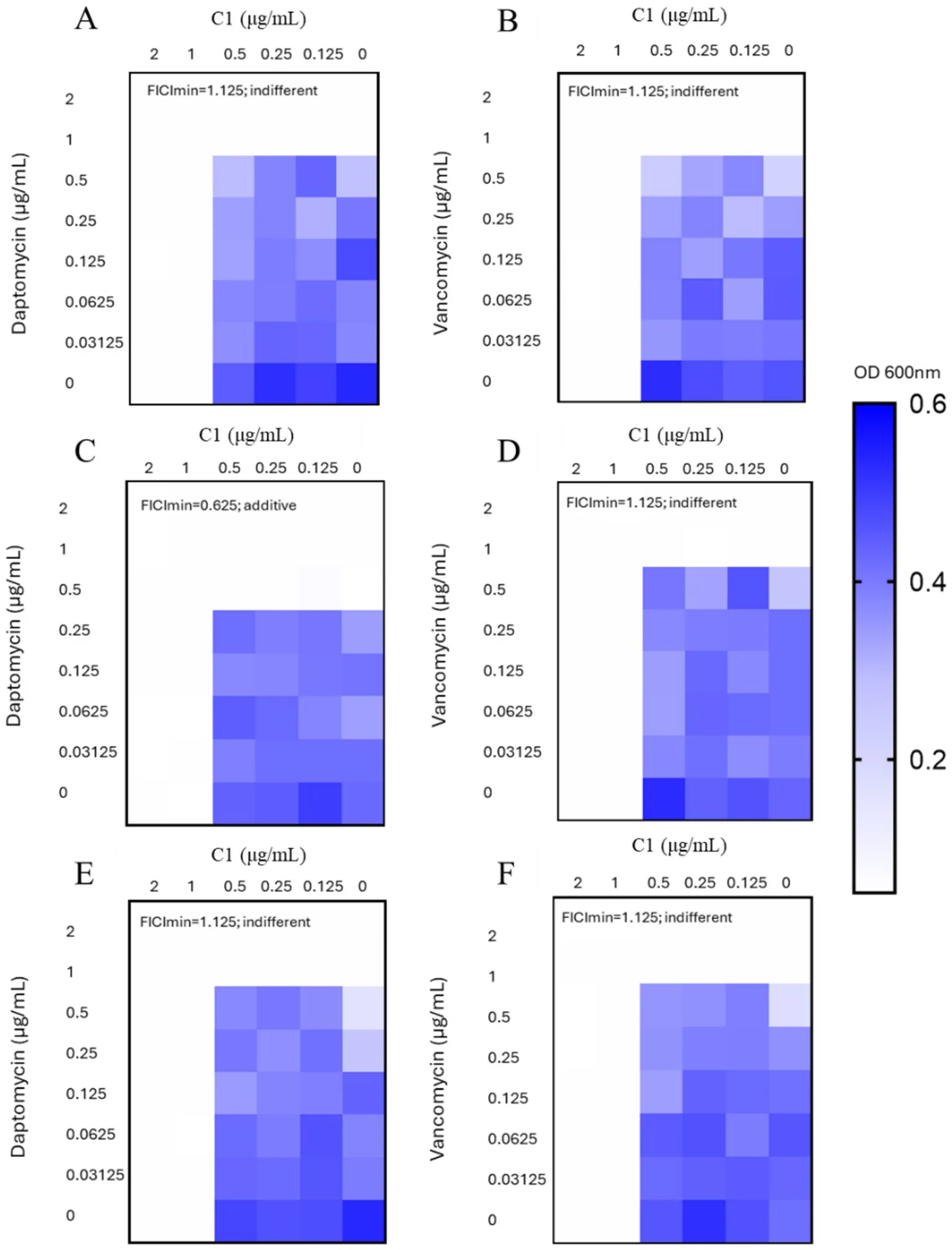
Effects of combination treatment with compound **1** and daptomycin or vancomycin on clinically relevant MRSA strains. Heatmaps depict the outputs of the checkerboard assays used for evaluating antibacterial drug interaction. Each square represents quantitative data expressed as the mean value of optical density (OD_600 nm_, *n* = 3). Dark blue regions and white regions represent highest and lowest bacterial growth densities, respectively. **(A,B)** Clinical isolate D15 (CA-MRSA); **(C,D)** clinical isolate D17 (HA-MRSA); **(E,F)** reference MRSA strain USA300. The FIC index (FICI) was calculated as the sum of the individual FICs of the antibacterial compounds under test in each well. FICI values were interpreted as follows: synergistic (Syn = FICI ≤ 0.5); additive (Add = 0.5 < FICI ≤1); indifferent (Ind = 1 < FICI ≤ 4); or antagonistic effect (Ant = FICI >4).

To assess the potential toxic of compound **1**, we first evaluated its hemolytic activity against human red blood cells (Supplementary Figure S15A). The concentration causing 50% hemolysis (HEC_50_) was 34.54 μg/mL, while daptomycin and vancomycin showed no hemolysis up to 200 μg/mL. Notably, the HEC₅₀ of compound **1** is about four times higher than its MBC against MRSA and MSSA strains, indicating moderate hemolysis (~23%) at the bactericidal concentration of 8 μg/mL. Compared to other antibacterial secondary metabolites, the hemolytic activity of compound **1** at this concentration is considered moderate (Saleem et al., 2024).

We further evaluated the effect of compound **1** on the metabolic activity of human bronchial respiratory epithelium cells (16HBE14o-) as a mammalian-cell cytotoxicity assessment (Supplementary Figure S15B). Compound **1** affected the cell metabolic activity of 16HBE14o- cells in a dose-dependent manner, with an IC_50_ of 2.39 ± 0.31 μg/mL. In comparison, daptomycin and vancomycin showed (IC_50_ values of 111.25 ± 12.86 μg/mL and > 200 μg/mL), respectively. Thus, compound **1** exhibited substantially greater cytotoxicity toward 16HBE14o- cells than the two reference antibiotics under the conditions tested.

Previous studies have reported moderate cytotoxicity of CJ-21058 against HeLa cells, with an IC_90_ of approximately 32 μg/mL (Sugie et al., 2002). However, direct quantitative comparison between the previously reported HeLa-cell IC_90_ and the present IC_50_ value obtained in 16HBE14o- cells is limited by differences in cell type and assay endpoint.

Taken together, compound **1** exhibited antibacterial activity accompanied by measurable hemolytic activity and mammalian-cell cytotoxicity. The IC_50_ value obtained in 16HBE14o- cells was lower than its antibacterial MBC, indicating a limited selectivity window in this mammalian-cell model. Therefore, further investigation and structural optimization will be required to improve the selectivity of compound 1 before it can be considered a promising antibacterial lead.

### 3.5 Genome analysis of *Neocucurbitaria* sp. VM-36 suggests the potential to produce a diverse array of antibacterial small molecules

Based on Gene Ontology analysis, the top 50 terms are categorized into biological processes (24%), molecular functions (52%), and cellular components (24%) (Supplementary Figure S16A). Among these, carbohydrate metabolism was prominently represented, with 224 genes annotated under “carbohydrate metabolic process”, 694 under “hydrolase activity”, and 129 under “pectate lyase activity”. More in-depth analysis revealed the presence of 571 carbohydrate-active enzyme-related genes. This highlights the ecological role and biotechnological potential of *Neocucurbitaria* sp. VM-36 in plant biomass decomposition and carbohydrate metabolism. This potential appears to be shared with other Cucurbitariaceae species (Supplementary Figure S16B), suggesting conserved metabolic strategies likely shaped by shared ecological niches and evolutionary pressures.

To explore the biosynthetic potential of *Neocucurbitaria* sp. VM-36, we searched its genome and those of six other strains in the Cucurbitariaceae family for putative biosynthetic gene clusters (BGCs) and compared them. A total of 34 putative BGCs were detected in *Neocucurbitaria* sp. VM-36, a number comparable to the average BGC count (36) across the other Cucurbitariaceae fungi (Figure 6A and Supplementary Table S6). Based on core gene annotations, these BGCs are putatively categorized into five major types: 10 polyketide synthase (PKS)-type BGCs, 9 ribosomally synthesized and post-translationally modified peptide-like BGCs, 9 non-ribosomal peptide synthetase (NRPS) and NRPS-like BGCs, 3 terpene-type BGCs, and 3 hybrid BGCs (2 NRPS+T1PKS and 1 T1PKS + fungal-post-translationally modified peptide-like). Annotation against the MIBiG database revealed that 11 of the 34 BGCs showed similarity to previously characterized BGCs (Figure 6B and Supplementary Figure S17). Notably, BGCs with similarity to clusters associated with choline and metachelin C biosynthesis were conserved across all seven analyzed fungi, suggesting a common biosynthetic capability within the family. Additionally, BGCs showing similarity to those associated with squalestatin S1, secalonic acids, scytalone/T3HN, (−)-mellein, and phyllostictin A biosynthesis were identified not only in *Neocucurbitaria* sp. VM-36 but also in select species within the Cucurbitariaceae family. Importantly, four BGCs, putatively associated with the biosynthesis of metabolites related to the antifungal agent griseofulvin, the antibacterial agents usnic acid and phomasetin, and the anticancer agent hypothemycin, were found only in *Neocucurbitaria* sp. VM-36. These unique clusters suggest a distinct biosynthetic repertoire, with the potential to contribute to the production of structurally and biologically diverse metabolites (Supplementary Information Note S1).

**Figure 6:**
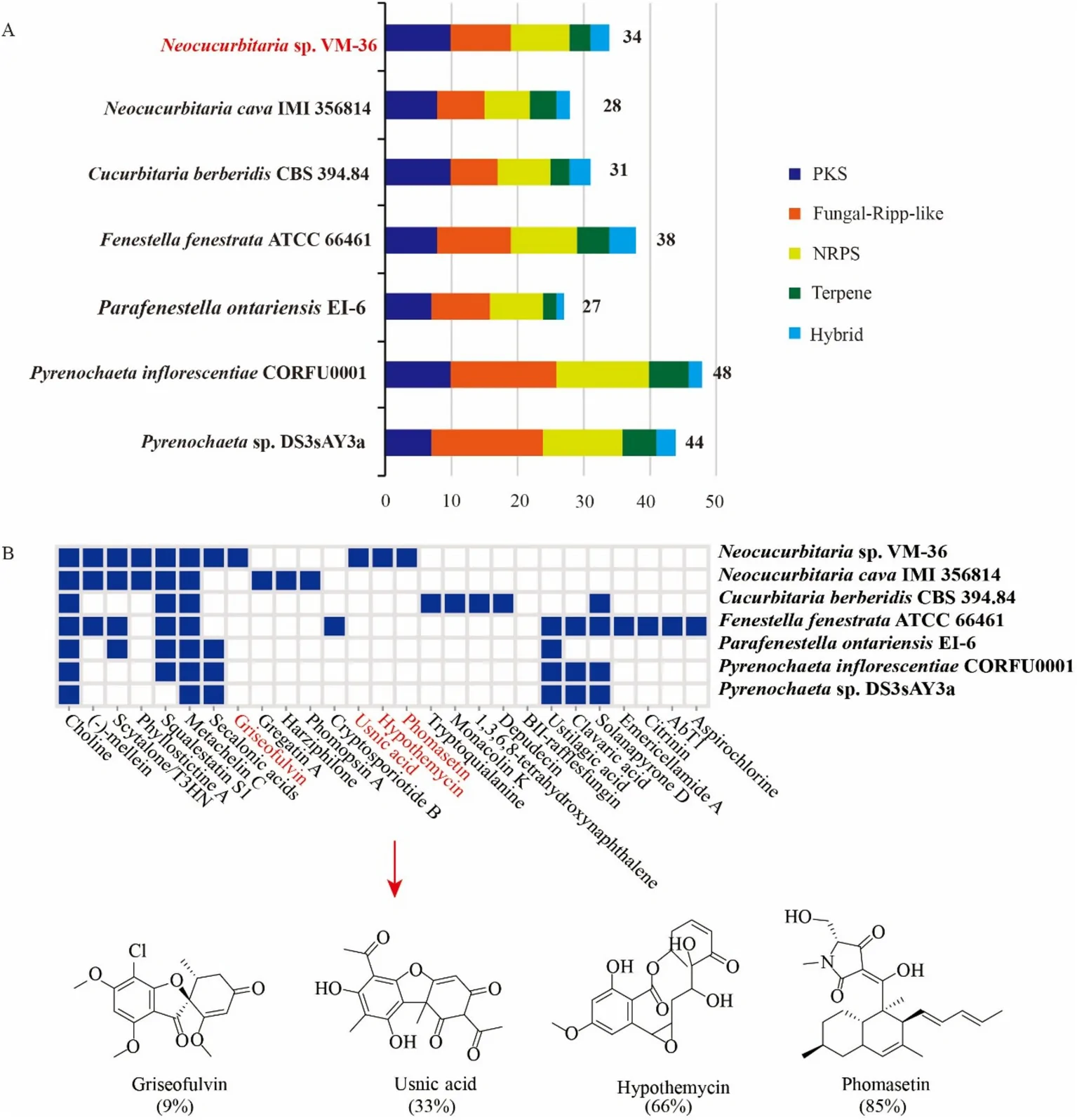
Comparative analysis of predicted BGCs in 7 strains of the Cucurbitariaceae family. **(A)** Number of BGCs in each genome by BGC class; **(B)** Presence (blue) or absence (white) of those BGCs with similarity matches in the MiBIG database; chemical structures of metabolites correlated with the BGCs highlighted in red according to the MiBIG database are shown at the bottom, with the BGC similarity score (*Neocucurbitaria* sp. VM-36 versus MiBIG reference entry) shown in percent.

### 3.6 Metabolomic profiling reveals a diverse metabolite repertoire in *Neocucurbitaria* sp. VM-36

So far, only 17 compounds have been isolated from *Neocucurbitaria* strains, encompassing macrocyclic and phomactin diterpenes and their derivates (Hu et al., 2022a, 2022b; Liu et al., 2026), and a total of 30 compounds have been isolated from the Cucurbitariaceae family (Supplementary Table S7 and Supplementary Figure S18). The limited number of reports on secondary metabolites (SMs) from this family aroused our interests for exploring the chemical repertoire of *Neocucurbitaria* sp. VM-36 and investigate their putative biosynthetic origins. Thus, we performed untargeted metabolomic analysis of fungal crude extracts from PDA and DPY media, together with corresponding medium blank controls. The resulting molecular network consists of 1,521 nodes and 1,896 edges (Figure 7 and Supplementary Table S2). In total, 19 compounds are annotated based on library matching against MS/MS databases, and 7 compounds are putatively assigned based on their relationships to matched adjacent nodes (Figures 7, 8).

**Figure 7:**
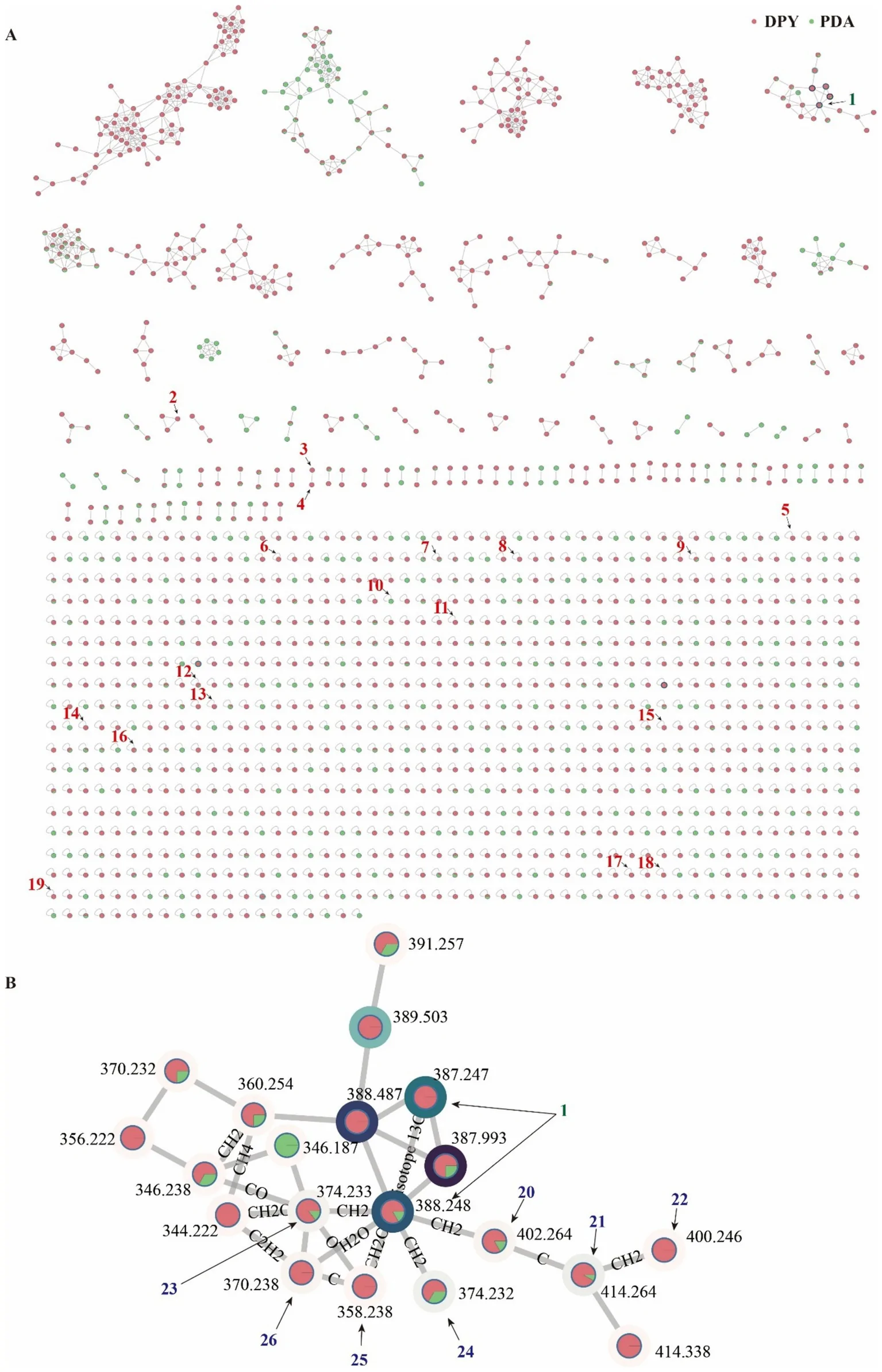
Molecular network of mass spectrometric features detected in crude EtOAc extracts of *Neocucurbitaria* sp. VM-36 grown in PDA solid medium and DPY liquid medium. **(A)** Overview of the molecular network, with putatively annotated SMs; **(B)** Molecular network of putative tetramic acid compounds containing decalin rings (cluster 5). Node colors represent signals in samples from PDA solid medium (green) and DPY liquid medium (red). Darker shades of node borders indicate higher metabolite abundance in samples based on peak area integration of the base peak, and purple represents the highest abundance. Edges represent the structural similarity between nodes. The annotations adjacent to the network edges (e.g., CH_2_, CO, H_2_O) represent putative neutral mass differences derived from the observed *m/z* shifts between connected nodes. The numbers (**1**–**26**) adjacent to the nodes are color-coded according to the confidence level of structural annotation: green indicates isolated compound; red represents putative annotations based on spectral library matching; blue denotes putative compound class annotations inferred from matched adjacent nodes in the molecular networking. Each node represents a molecular feature.

**Figure 8:**
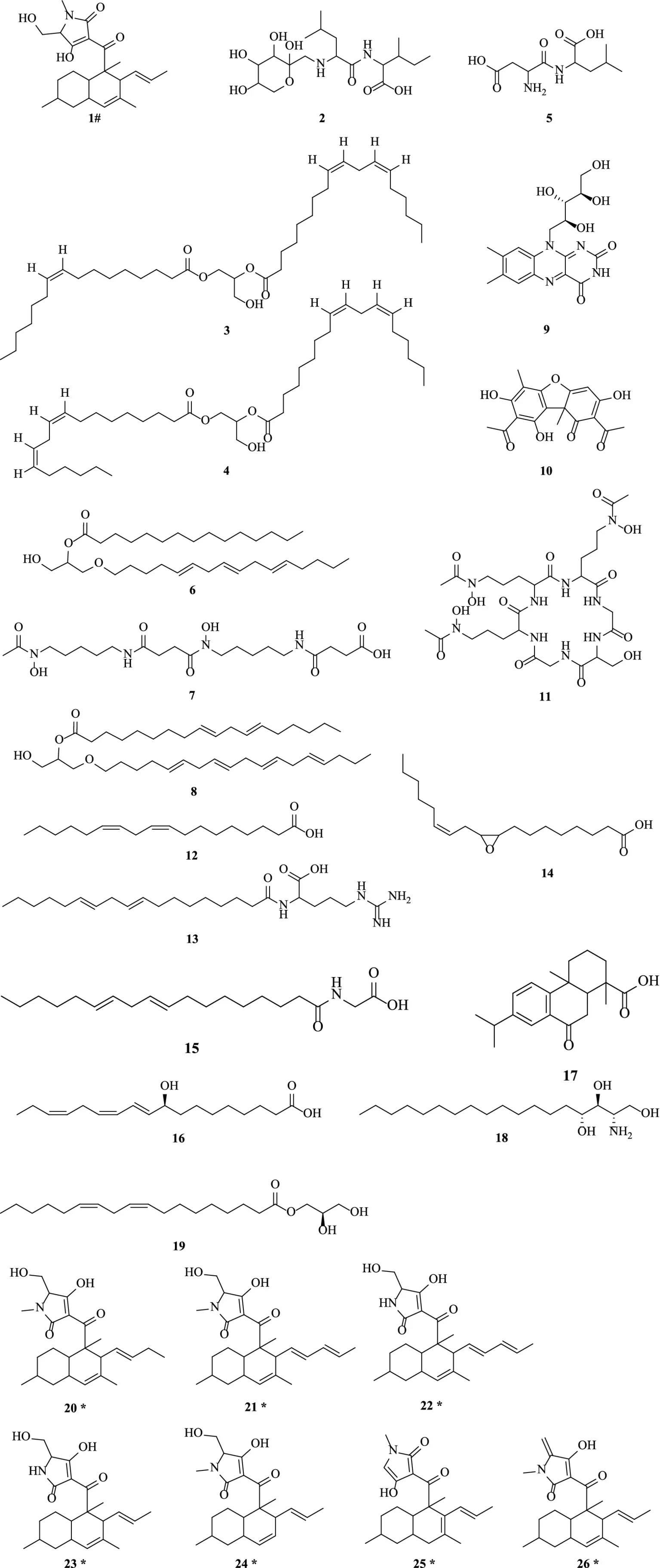
Chemical structures of putatively annotated compounds in the crude extracts of *Neocucurbitaria* sp. VM-36 grown on PDA solid medium or DPY liquid medium. Compounds confirmed by spectroscopic analysis are marked with a hash symbol (#); compounds putatively annotated via spectral library matching are shown without special markers; and DTAs putatively annotated based on shared spectral similarities and neutral losses with respect to compound **1** are marked with an asterisk (*).

The major constituents with high abundance in the crude extracts are annotated as CJ-21058 analogues in cluster 5 (Figure 7B), representing tetramic acid-type metabolites containing decalin rings, i.e., decalin-containing tetramic acids (DTAs). Based on the observed MS/MS fragmentation patterns (Supplementary Table S2), 7 additional tetramic acid-type metabolites are putatively assigned (Supplementary Information Note S2).

Among the less abundant compounds, a total of 9 lipids, including compounds **3** (DG(16:1/18:2/0:0)), **4** (DG(18:2/0:0/18:2)), **6** (AEG(o-16:3/15:0)), **8** (AEG(o-18:4/18:2)), **12** (linoleic acid), **14** (9(10)-EpOME), **16** (9S-Hydroxy-10*E*, 12*Z*, 15*Z*-octadecatrienoic acid), **18** (phytosphingosine), and **19** (1-linoleoylglycerol), are annotated as singleton nodes in the molecular network. Six amino acid-containing or peptide compounds, including compounds **2** (FruLeuIle), **5** (Asp-Leu), **7** (4-[5-[[4-[5-[acetyl(hydroxy)amino]pentylamino]-4-oxobutanoyl]-hydroxyamino] pentylamino]-4-oxobutanoic acid), **11** (N-[3-[5, 8-bis[3-[acetyl(hydroxy)amino]propyl]-14-(hydroxymethyl)-3, 6, 9, 12, 15, 18-hexaoxo-1, 4, 7, 10, 13, 16-hexazacyclooctadec-2-yl]propyl]-N-hydroxyacetamide/ desferriferricrocin), **13** (Arg-C18:2), and **15** (Gly-C18:2), are also found scattered across the network. Compound **11** is a cyclic hexa-peptide hydroxamate siderophore, with the general formula RC(O)N(OH)R′ (*N*-hydroxy amides) (Zhao et al., 2022). Compounds of this type do not only help fungi to chelate iron for survival under iron-restricted conditions, but they have also attracted attention due to their medicinal value as potent antibacterial or antitumor agents (Zhao et al., 2022). In *Neocucurbitaria* sp. VM-36, we hypothesize that the BGC encoded in region 8.2 (hybrid BGC of NRP-metallophore and NRPS) is likely to be a candidate BGC potentially associated with compound **11**. Although there are no results with Known-Cluster-Blast, the core NRPS gene in the BGC shows amino acid similarity with an epichloenin A synthetase (MIBIG protein-AET13875.1, BGC0001250), with 26% identity and 82.7% coverage. Epichloenin A is an extracellular siderophore required to maintain the mutualistic interaction between the endophyte *Epichloe festucae* and perennial ryegrass (*Lolium perenne*) (Johnson et al., 2013). Specifically, secretion of epichloenin A enables the symbiotic fungus to compete for plant iron while avoiding fungal over-growth inside the host plant (Johnson et al., 2013). Moreover, another core gene in the NRP-metallophore BGC of *Neocucurbitaria* sp. VM-36 shares similarity with an *L*-ornithine_N-monooxygenase (MIBiG protein CAJ96465.1) from BGC0000330, with 42% amino acid identity and 87% coverage, supporting its putative involvement in the formation of an N-hydroxylated and N-acylated ornithine moiety in compound **11** (Kreutzer et al., 2012).

Node **10** is predicted to correspond to the [M + H + CH_3_OH]^+^ adduct of isousnic acid, an isomer of usnic acid. The cluster in region 20.2 in *Neocucurbitaria* sp. VM-36 has a Known-Cluster-Blast hit with BGC0002483, a characterized usnic acid BGC, and 33% of its encoded enzymes show amino acid similarity to the query sequences. In particular, it encodes enzymes similar to the non-reducing PKS (methylphloracetophenone synthase) and a cytochrome p450 (methylphloracetophenone oxidase), which are involved in usnic acid biosynthesis in the reference pathway (Abdel-Hameed et al., 2016). Usnic acid, a toxic dibenzofuran, has been discovered in lichens and was extensively reported to have a variety of pharmacological effects, including remarkable antimicrobial, antitumor, antiviral, and antiparasitic activities (Macedo et al., 2021; Sepahvand et al., 2021). It is active against Gram-positive bacteria, such as *B. subtilis* and *S. aureus*, by inhibiting RNA and DNA synthesis and blocking DNA replication and elongation, but it is not active against Gram-negative bacteria (Sepahvand et al., 2021). Although usnic and isousnic acid are regioisomers, the latter is comparatively poorly investigated, with only a few studies reporting on its antimicrobial and anti-inflammatory properties (Goel et al., 2011; Mendili et al., 2022).

Overall, many clusters in the molecular network, especially those containing three or more nodes, could not be confidently assigned to specific compound classes or structures. These features may represent previously uncharacterized metabolites and warrant further investigation through compound isolation and structural characterization.

### 3.7 Proposed biosynthetic pathway for compound **1**

The cluster in region 13.1, classified as a hybrid T1PKS/NRPS BGC, shares partial similarity with the phomasetin biosynthetic gene cluster from *Pyrenochaetopsis* sp. RK10-F058 (BGC0001738). Therefore, we propose it as a candidate BGC potentially associated with the biosynthesis of compound **1** (Figure 9). In particular, the core enzyme NeoA shares 72% amino acid identity with the PKS-NRPS hybrid Phm1 in this cluster (Kato et al., 2018). It comprises one highly-reducing PKS module and one NRPS module with a terminal reductase domain. The PKS module is likely complemented by a stand-alone enoyl reductase (NeoB), potentially enabling formation of a linear polyene intermediate via 7 cycles of polyketide chain elongation, reduction, and methylation. We propose that *L*-serine is subsequently incorporated by the NRPS module and that upon release from the assembly line the tetramic acid moiety is formed through the Diekmann cyclase function of the terminal reductase domain, as described for phomasetin biosynthesis (Kato et al., 2018). We further hypothesize that NeoC, displaying 57% amino acid identity with Phm7 (Kato et al., 2018; Chi et al., 2021; Fujiyama et al., 2021), functions as the Diels-Alderase catalyzing the intramolecular cycloaddition to form the cyclohexene ring (Fujiyama et al., 2021). We furthermore propose that NeoD, sharing 71% amino acid identity with the methyltransferase Phm5, may catalyze N-methylation of the tetramic acid moiety of an N-demethylated precursor. This precursor could be compound **23** in our molecular network based on its spectral similarity to compound **1**.

**Figure 9:**
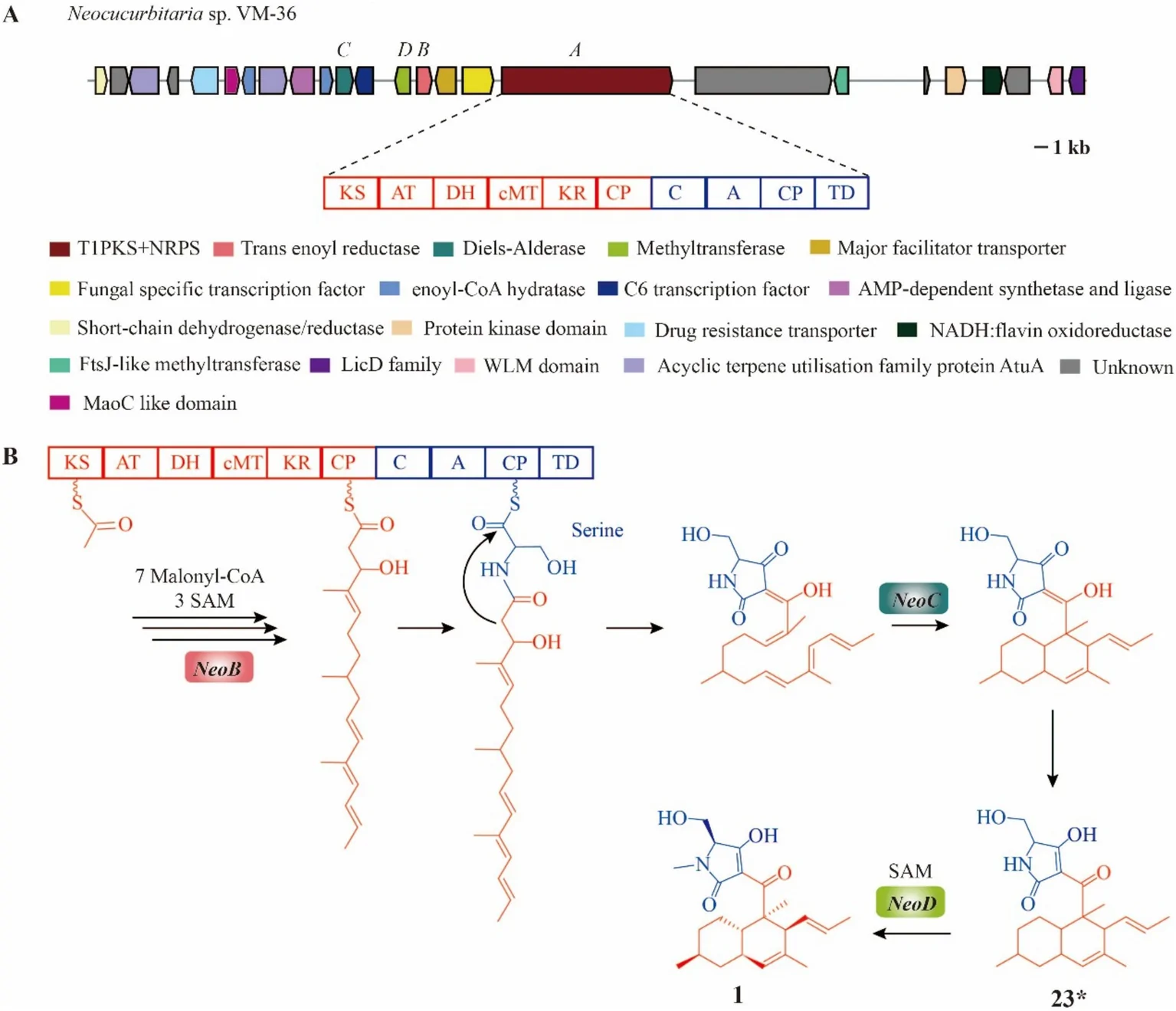
Proposed gene cluster **(A)** and biosynthetic pathway **(B)** for compound **1**. β-ketosynthase (KS), acyl-transferase (AT), dehydratase (DH), carbon-methyltransferase (cMT), β-ketoreductase (KR), and acyl-carrier protein (ACP), condensation (C), adenylation (A), peptidyl carrier protein (PCP), and terminal reductase (TD).

## 4 Discussion

*Staphylococcus aureus*, an opportunistic pathogen producing virulence factors like hemolysins and leukocidins, remains a major clinical challenge (Jin et al., 2024). Antibiotic overuse has driven the rise of drug-resistant strains, especially MRSA and other ESKAPE(E) pathogens, leading to frequent failures in the treatment of bacterial infections. Therefore, the pursuit of effective antibacterial agents has garnered significant interest. In this study, we investigated *Neocucurbitaria* sp. VM-36, an endophytic fungus whose biosynthetic potential and antibacterial properties have not been previously characterized. Although decalin-containing tetramic acids (DTAs) have been reported from several fungal genera, their occurrence in *Neocucurbitaria* species has not previously been documented. Through integrated genomic, metabolomic, and antibacterial analyses, we provide evidence for the occurrence of DTA-related metabolites in *Neocucurbitaria* sp. VM-36, while compound **1** was identified as a major antibacterial metabolite.

Compound **1** exerts strong antibacterial effects on Gram-positive bacteria, while showing no inhibitory effects against Gram-negative bacteria, consistent with the antibacterial spectrum commonly reported for DTAs (Kim et al., 2024). DTAs have attracted considerable attention due to their potent activities against diverse bacterial pathogens, including *B. subtilis*, MRSA, vancomycin-resistant *Enterococcus*, and multidrug-resistant *Mycobacterium tuberculosis* (Whitt et al., 2014; Gao et al., 2024; Kim et al., 2024). Compared with previously reported DTAs against MRSA, which showed MIC values ranging from 0.002 to 42 μg/mL, compound **1** shows excellent antibacterial efficacy, with MIC values of 0.5–1 μg/mL (Kim et al., 2024). In comparison to equisetin (MIC = 1.25 μg/mL) (Whitt et al., 2014), the presence or absence of the methyl group at the C-4 position, as well as differences in stereo-centers, do not appear to significantly affect the antibacterial activity against the tested MRSA strains. Recent research has explored the structure–activity relationship of the antibacterial activity of DTA derivatives against MRSA, identifying some key chemical features associated with antibacterial potency (Kim et al., 2024). For instance, the dehydrogenation of the tyrosine sidechain in the tetramic acid substructure of conipyridoins, and the stereochemical configuration at the C-5’ position in 5’-epi-equisetin are crucial for their antibacterial activity against MRSA (Kariya et al., 2023; Kim et al., 2024). Additionally, for certain DTAs, such as *N*-demethylophiosetin, paecilosetin, and 8-epi-equisetin, the absence of oxygen groups and the stereochemistry of the methyl groups at the C-8 position on the decalin skeleton are important determinants of their antibacterial efficacy (Kariya et al., 2023; Kim et al., 2024). In cordysetin A, the *D* configuration of serine and *N*-methylation of the tetramic acid are crucial for its anti-tuberculosis activity (Gao et al., 2024). Conversely, the antifungal activity of fusaramin appears to be more dependent on the identity of the amino acid than its cyclization to tetramic acid (Kimishima et al., 2024). Collectively, these findings indicate that subtle structural variations within the DTA scaffold can markedly influence biological activity, highlighting the complexity of the structure–activity relationships within this class of compounds.

Our results demonstrate that compound **1** exhibits potent bactericidal activity against *S. aureus* strains USA300, D15, D17 (MRSA), and ATCC 29213 (MSSA), outperforming daptomycin and vancomycin at 8 μg/mL via a tentative bacteriolytic like mechanism. Further tests, such as membrane-permeability assays or the analysis of leakage of intracellular components, are needed to validate the bacteriolytic-like mechanism. Additionally, compound **1** showed an additive effect with daptomycin against the clinical isolate *S. aureus* D17 (FICI_min_ = 0.625). Combination therapy is commonly used to treat infections caused by multidrug-resistant bacteria, as it broadens the antibacterial spectrum, lowers resistance development, and reduces adverse effects (Herrera et al., 2022). Previously, the DTA compound equisetin has been demonstrated to effectively eradicate MRSA or VRE with low-level antibiotic resistance and to synergize with colistin, although it had indifferent effects with other antibiotics, including daptomycin (Chen et al., 2021). Additionally, equisetin can efficiently eliminate intracellular *S. aureus* by enhancing host autophagy and inducing mitochondria-mediated reactive oxygen species production, displaying notable anti-infective activity in a peritonitis mouse model (Tian et al., 2022). Given the structural similarity, compound **1** may have related antibacterial properties. However, its molecular target and mode of action require further investigation.

Although compound **1** demonstrated promising *in vitro* antibacterial activity, its cytotoxicity toward 16HBE14o- cells and moderate hemolytic activity represent important limitations that warrant further investigation, particularly in the context of potential systemic applications. Previous studies have also reported moderate cytotoxicity of CJ-21058 against HeLa cells at concentrations corresponding to an IC_90_ of approximately 32 μg/mL (Sugie et al., 2002). However, differences in cell type and assay endpoint preclude direct quantitative comparison with the present results. Previous studies have explored localized delivery strategies for related DTA compounds, including wound dressings, microneedles, and electrospun nanofibers for equisetin (Luo et al., 2018; Xing et al., 2025; Yedavally et al., 2026). These approaches may provide useful directions for future investigation. However, their applicability to compound **1** remains unknown, and proposing a specific administration route would therefore be premature. Further studies on stability, solubility, pharmacokinetics, resistance development, and *in vivo* efficacy and safety are needed to better assess its therapeutic potential.

Previous investigations of *Neocucurbitaria* have mainly focused on ecological characterization, with only limited studies exploring its secondary metabolites, primarily diterpenoids from *N. unguis-hominis* FS685 (Hu et al., 2022a, 2022b; Liu et al., 2026). In this study, the integrated genomic, metabolic, and antibacterial analyses of *Neocucurbitaria* strain provide new insights into its biosynthetic potential. Genome mining revealed multiple predicted biosynthetic gene clusters, while metabolomic analysis indicated the occurrence of DTA-related and other specialized metabolites. Together with the identification of compound 1 as an antibacterial metabolite, these findings expand the current knowledge of the chemical and biosynthetic potential of *Neocucurbitaria* and support further investigation of this understudied genus.

## 5 Conclusion

This study identifies *Neocucurbitaria* sp. VM-36 as an underexplored endophytic fungus with potential for antibacterial natural product discovery. Compound **1** exhibited strong bactericidal activity against both MSSA and MRSA strains, highlighting its potential as a promising scaffold for further development against Gram-positive bacterial pathogens. Integrated genomic and metabolomic analyses further revealed a diverse repertoire of BGCs and multiple additional MS features that were putatively annotated as structurally related metabolites. Among these, a candidate BGC was proposed to be associated with the biosynthesis of compound **1**. However, the identities of the putatively annotated metabolites and the proposed BGC-metabolite relationship require further structural and genetic validation. Overall, these findings highlight *Neocucurbitaria* sp. VM-36 as a promising source for further investigation of fungal antibacterial natural products.

## Data Availability

The datasets presented in this study can be found in online repositories. The names of the repository/repositories and accession number(s) can be found in the article/Supplementary material.

## Footnotes

1
https://genome.jgi.doe.gov/portal/

2
https://www.ncbi.nlm.nih.gov/

3
https://www.rdkit.org/

4
https://www.gensas.org

5
https://github.com/tseemann/barrnap

6
https://gnps.ucsd.edu/

7
https://gnps.ucsd.edu/ProteoSAFe/status.jsp?task=9099def02462433487a26b71afb487e4

## Glossary

ATCC: American type culture collection
BGCs: biosynthetic gene clusters
BLAST: basic local alignment search tool
CD: circular dichroism
CFUs: colony-forming units
DMSO: dimethyl sulfoxide
DPY: dextrose peptone yeast
DTAs: decalin-containing tetramic acids
ECD: electronic circular dichroism
EtOAc: ethyl acetate
FICI: fractional inhibitory concentration index
GNPS: global natural products social molecular networking
IC_50_: 50% inhibitory concentration
IC_90_: 90% inhibitory concentration
ITS: internal transcribed spacer
MBC: minimum bactericidal concentration
MEA: malt extract agar
MICs: minimum inhibitory concentrations
MRSA: methicillin-resistant *Staphylococcus aureus*
MSSA: methicillin-sensitive *Staphylococcus aureus*
NCTC: national collection of type cultures
NMR: nuclear magnetic resonance spectroscopy
NRPS: non-ribosomal peptide synthetase
PDA: potato dextrose agar
PKS: polyketide synthase
SDA: sabouraud dextrose agar
SMs: secondary metabolites
SNA: synthetically nutrient-poor agar
