# Supplementary material for: Genomics and metabolomics analyses reveal the endophytic fungus Neocucurbitaria sp. VM-36 as a potential source of bioactive metabolites against methicillin-resistant Staphylococcus aureus
Source: Front Microbiol. 2026 Oct 15;17:1929829. doi: 10.3389/fmicb.2026.1929829 (PMC13716798; doi:10.3389/fmicb.2026.1929829)
Supplement: Supplementary file 1 [file Supplementary_file_1.DOCX]

Supplementary Material

## 1. Supplementary Methods

### 1.1 Antibacterial susceptibility assays

#### 1.1.1 Minimum Inhibitory Concentrations (MICs) and Minimum Bactericidal Concentrations (MBCs) of compound 1

The broth microdilution method was used to determine the MIC values of compound **1** against different bacterial strains according to the guidelines from the European Committee for Antibacterial Susceptibility Testing (The European Committee on Antimicrobial Susceptibility Testing [EUCAST], 2022). In brief, compound **1** was solubilized in DMSO, filtered with a 0.22 µm PTFE membrane, and serially diluted in sterile double-distilled water to obtain a concentration range of 0.03 - 64 µg/mL. Bacteria were reactivated on cation adjusted Mueller Hinton (MH) agar (Bioxon®) plates for 24 h at 36 ± 1º C. Overnight cultures were then prepared in cation adjusted MH broth at 36 ± 1 ºC with constant shaking at 250 revolutions per min (rpm), and added to 96-well microtiter plates at a final concentration of 5 $\times$ 10^4^ colony-forming units (CFUs)/well. Internal controls were used for each assay and included medium containing 10% DMSO (solvent control), medium inoculated with bacterial cells (bacterial growth control), and medium only (medium control). The final concentration of DMSO in the assay was ≤ 0.6% *v*/*v*_final_. Plates were incubated in a Biotek® Epoch-2 microplate reader at 36 ± 1 ºC for 24 h with double-orbital agitation at a frequency of 282 cycles/min. The values of the lowest concentration of compound **1** that completely inhibited bacterial growth were determined as the MICs. The MIC values of ESKAPE strains were firstly evaluated (Table S5).

Due to the strong antibacterial effect of compound **1** on MRSA strains, we further determined the MBC values of this compound against 4 MRSA strains, namely *S. aureus* USA300, D15 and D17, and NE1688 (Sle1). The *S. aureus* ATCC 29213 strain was also included as a MSSA reference. The glycopeptide vancomycin and the cyclic lipopeptide daptomycin were used as positive controls. A volume of 2.5 µL of bacterial broth was taken from the MIC assay wells, with corresponding concentrations of 4 $\times$MIC, 2 $\times$MIC, and MIC, and then used to inoculate MH agar plates for MBC determination upon incubation at 36 ± 1 ºC for 24 h. Bacteria treated with the respective vehicles for compound **1**, vancomycin, and daptomycin served as negative controls (DMSO ≤0.6% v/v_final,_ double-distilled water, and NaCl 0.9% w/v, respectively). The MBC was determined as the lowest concentration of compound **1** that reduced the viable bacterial count on the agar plates by 99.9%. All tests were performed in triplicates.

#### 1.1.2 Bacterial growth assay after compound 1 treatment

To monitor the effects of compound **1** on different MRSA and MSSA strains growth experiments were performed, using vancomycin and daptomycin as controls. In brief, the strains were reactivated, cultured, and seeded in 96-well microtiter plates as described above. After 10 h incubation, bacteria were treated with compound **1** or the control antibiotics at final concentrations of 2, 4, and 8 µg/mL, and incubated at 36 ± 1 ºC. Bacteria treated with the respective vehicles for compound **1**, vancomycin, and daptomycin served as negative controls (DMSO ≤ 0.6% v/v_final,_ double-distilled water, and NaCl 0.9% w/v, respectively). All tests were performed in triplicate (n = 3). The bacterial growth was monitored by OD_600_ measurements at 20 min intervals and results are presented using Graphpad^®^. The results are expressed as follows:

$$Bacterial growth \left( \mathrm{OD}600 \right)= mean OD600 value of compound treated or untreated bacteria - mean OD600 value of vehicle treated bacteria$$

The viability of bacteria was measured before (t = 8 h) and after 40 h compound exposure (t = 48 h). Briefly, 2 µL of bacterial broth was taken from each well, diluted (1: 10^6^ dilution factor), and then 2.5 µL of the dilutions were seeded onto MH agar plates, which were incubated at 36 ± 1 ºC for 24 h before CFUs were counted. The amount of CFUs/mL was calculated.

### 1.2 Hemolysis assay with human erythrocytes

Human red blood cells (hRBCs) were isolated from approximately 10 mL of whole blood from healthy donors using Lymphoprep buffer^TM^ (Stem cell technologies, Canada) following the manufacturers protocol with some modifications in the centrifugation parameters (1500 rpm, 30 min, 19 ℃). The fraction corresponding to hRBCs and neutrophils (aprox. 4 mL) was washed with 1 mL of 150 mM NaCl by centrifugation at 500$\times$ g, 5 min and 20 °C. Then, stock solutions of hRBCs at 2% (v/v) hRBCs were preserved at 4 °C for a maximum of 4 weeks. These were made by mixing 1 mL of the fraction from the previous stem with 49 mL of modified ‘saline adenine glucose mannitol’ (SAGM, final pH 6.1) medium (Zehnder et al., 2008) prepared in Dulbecco’s phosphate-buffered saline (DPBS) buffer. hRBCs were allowed to precipitate in the storage media for 48 h before using them for bioassays.

On the day of experiments, the hRBCs stock was washed three times with DPBS (pH 7.2) by centrifuging (500$\times$ g, 5 min, 20 °C) to remove the SAGM medium. Subsequently, the hRBCs were resuspended in DPBS. 180 µL of a 1% (v/v) solution of intact hRBCs in DPBS (pH 7.2) were loaded into the wells of a U-shaped, 96-well microplate Greiner®. Then the hRBCs were treated with four concentrations of compound **1** (final in-test concentrations 200, 100, 10, 1 µg/mL). Also, four concentrations of the compound’s vehicle (DMSO at in-test concentrations 1%, 0.5% 0.05% and 0.005% v/v) were evaluated to be used as blanks. Treatment of hRBCs with a solution of sodium dodecyl sulfate at a final in-test concentration 0.1% w/v was used as positive control for hemolysis, whereas addition of DPBS pH 7.2 served as negative control. Vancomycin and daptomycin were included in the evaluation for comparative purposes at the same final in tests concentrations as used for compound **1**. Their vehicles, double-distilled water or NaCl 0.9% w/v, respectively, were also evaluated as their blanks. Afterwards, the plates containing treated hRBCs were incubated at 37 °C for 24 h, and the microplate was then centrifuged at 500 rpm (10 min, 26 °C). Subsequently, 50 µL of the supernatant from the treated hRBCs were transferred into a new flat-bottomed 96-well plate containing 150 µL of DPBS pH 7.2. To quantify hemolysis, the plate was read at an optical density of 410 nm (OD_410 nm_) in a Biotek® instrument. The hemolytic activity was described in terms of percentage of hemolysis (% of hemolysis) and was calculated considering the Standard Practice for Assessment of Hemolytic Properties of Materials (ASTM F-756-00), using a previously reported equation (Wang et al., 2016) adapted to our assay conditions as:

$Percentage of hemolysis \left( \% of hemolysis \right)=100 \times(OD410nm of compound treated hRBCs - OD410nm of vehicle treated hRBCs)/ (OD410nm of positive control - OD410nm of negative control)$.

The results were then reported as the arithmetic mean of the percentage of hemolysis ± standard deviation obtained from each treatment. Additionally, the concentration of the antibacterial compound capable of inducing a hemolytic effect in 50% of the hRBCs (HEC_50_) was obtained through linear regression analysis from 4-point regression plots obtained in Graphpad Prism® v. 8.0.1. Each treatment was tested in three technical replicates.

### 1.3 Cytotoxicity in human bronchial epithelial cells

The *in vitro* effect of compound **1** was also evaluated against immortalized cells from the human bronchial respiratory epithelium (16HBE14o-) (Cozens et al., 1994) via the resazurin assay. Cells were regularly maintained as previously described (Palma Medina et al., 2019) in supplemented eMEM medium (final pH 7.2) containing glutamine at 10 mM of (Capricorn Scientific®) and incubated at 37 °C in 5% CO_2_. Then 4000 cells/ well were seeded in flat bottomed, tissue culture treated, 96 well plates from Gibco® and incubated for 3 days before treatment with antibacterial compounds. After the incubation time, supplemented eMEM medium was renewed and four concentrations of the Compound **1** were tested (in-test concentrations 200, 100, 10, 1µg/mL). Also, four concentrations of the compound’s vehicle (DMSO at in-test concentrations 1%, 0.5% 0.05% and 0.005% v/v) were evaluated used as the respective negative controls. Vancomycin and daptomycin were included in the evaluation for comparative purposes at the same final in tests concentrations as the compound **1**. Double-distilled water used as main vehicle to deliver these compounds and evaluated as their respective negative control. Sodium dodecyl sulfate (SDS) at a final concentration of 0.1% w/v was used as positive control for inhibition of cell metabolic activity and treatment with eMEM media served as negative control. The microplates were incubated for 24 hours at 37 °C in 5% CO_2_. Afterwards, plates were washed with DPBS (twice) and fresh media added with a 1:10 v/v ratio of Presto Blue ® resazurin-based reagent (Invitrogen, Thermo Scientific®) was poured into to each well, following incubation for 90 min at 37 °C in 5% CO_2_. Sterile media without cells added with resazurin was used as blank. To finalize, cell viability was measured in terms of resofurin production, which acts is a fluorescent cell viability indicator (resulting from the reduction of resazurin by living cells). Fluorescence was measured at λ_excitation: 500/27_ and λ_emission: 620/10_ (50% top, 7 mm height, autogain) using a Synergy® microplate reader and recorded in terms of arbitrary fluorescence units (AFUs). The effect of the Compound **1**, vancomycin and daptomycin in the 16HBE14o- was described in terms of percentage of cell metabolic activity (Percentage of cell metabolic activity %) and was calculated using the following equation:

$$Percentage of cell metabolic activity (\%)=100 \times(AFUs mean value of compound treated cells - AFUs mean value of blank)/ (AFUs mean value of negative control treated cells- AFUs mean value of blank)$$

The results were then reported as the arithmetic mean of the percentage of cell metabolic activity ± standard deviation. Additionally, the half-maximal inhibitory concentration of the compounds capable of inhibiting 50% of the cell metabolic activity of 16HBE14o- cells (IC_50_) was obtained through linear regression analysis from 2-point regression plots. Results were plotted in Graphpad Prism® v8.0.1. Each treatment was tested in three technical replicates in two independent experiments.

## 2. Supplementary Results

### 2.1 Note S1. Comparative analysis and biosynthetic gene cluster (BGC) prediction

**Choline, metachelin, and scytalone/T3HN.** BGCs of *Neocucurbitaria* sp. VM-36 with choline (Region 3.3), metachelin C (Region 7.1), and scytalone/T3HN (Region 7.4) annotations are putatively associated with processes related to fungal growth and development.

Choline is suspected to play a vital role in the growth of filamentous fungi and the regulation of mycelial morphology (Markham et al., 1993). The cluster in region 3.3 of the *Neocucurbitaria* sp. VM-36 genome shows 100% identity to the reported choline-associated BGC from *Aspergillus nidulans* FGSC A4.

Metachelin C, on the other hand, is a coprogen siderophore, which is a small molecular iron chelator that participates in multiple cellular processes in fungi (Zhang et al., 2021). In *Metarhizium robertsii ARSEF* 23 growth in iron-deficient media was shown to lead to significantly up-regulated expression of two highly homologous genes *mrsidD* and *mrsidA* (NRPS core gene) and production of a series of coprogens and dimerumic acids, including metachelin C, metachelin A, metachelin A-CE, metachelin B, dimerumic acid 11-mannoside, and dimerumic acid (Zhang et al., 2021). These responses were proposed to relate to the maintenance of normal physiology and adaptation to adverse environments (Zhang et al., 2021). In the 7 *Cucurbitariaceae* fungi that we analyzed in this study, four genes, encoding an NRPS, an ABC transporter-related protein, a putative siderophore biosynthesis protein, and an AMP-dependent synthetase and ligase, are conserved and show high similarity to genes associated with metachelin C biosynthesis in *Metarhizium robertsii ARSEF* 23 (Zhang et al., 2021). In the *Neocucurbitaria* sp. VM-36 genome, these genes are located in region 7.1. These similarities suggest that region 7.1 may encode a siderophore-related biosynthetic pathway, although its association with metachelin C production remains to be experimentally validated.

Lastly, scytalone/T3HN are precursors for the biosynthesis of the fungal 1,8-dihydroxynaphthalene melanin pigment that plays important roles in fungal development, survival, colorization, UV protection, oxidative stress and pathogenesis, as well as virulence in plant and human hosts (Zhang et al., 2017, 2019; Zhu et al., 2021). The cluster in region 7.4 of the *Neocucurbitaria* sp. VM-36 genome exhibits 40% identity to the reported scytalone/T3HN-associated BGC from *Pestalotiopsis fici W106-1* and may be involved in the biosynthesis of these compounds. Notably, in our morphological characterization of *Neocucurbitaria* sp. VM-36, we observed that the fungus produces chlamydospores and yellow pigment upon 28 days of culture in SDA medium. Moreover, upon incubation for 77 days, black pigment was produced in high amounts, which was accompanied by the growth of hyphae, the production of conidia, and an increase in colony size. Thus, the region 7.4 BGC may potentially contribute to pigment biosynthesis and adaptation to biotic or abiotic stresses and may be associated with the observed morphological changes. However, these hypotheses require further experimental investigation.

**Xanthones.** Natural products with a xanthone scaffold are widely distributed in higher plants, lichens, bacteria, and fungi (Khattab and Farag, 2022; Veríssimo et al., 2022). Multiple substitution positions and the ability to multimerize render this class of compounds highly diverse and complex. Accordingly, it has attracted attention due to a broad array of bioactivities, including cytotoxic, antioxidant, antimicrobial, anti-fungal, anti-inflammatory, antithrombotic activities (Wezeman et al., 2015; Khattab and Farag, 2022; Veríssimo et al., 2022).

In the *Neocucurbitaria* sp. VM-36 genome, the cluster in region 13.3 shows similarity to a series of xanthone-related BGCs in the MIBiG database, including BGC0001886 (secalonic acids, 37% of genes show similarity), BGC0002063 (cryptosporioptide B, 23% of genes show similarity), BGC0001988 (neosartorin, 31% of genes show similarity), BGC0002726 (4-chloropinselin, 20% of genes show similarity), BGC0001403 (trypacidin, 28% of genes show similarity), BGC0000121 (RES-1214-2, 25% of genes show similarity), BGC0002592 (geodin, 33% of genes show similarity), BGC0002257 (rufoschweinitzin, 42% of genes show similarity), BGC0002244 (3’-methoxy-1,2-dehydropenicillide, 15% of genes show similarity), and BGC0002062 (agnestin A, 28% of genes show similarity). A common feature of these clusters is that the key non-reducing polyketide synthase (nr-PKS) can use acetyl and malonyl CoA to form C16 polyketides (Wezeman et al., 2015) and it does not carry a thioesterase (TE) domain. Therefore, an additional metallo-β-lactamase-type thioesterase is needed for releasing the polyketide from the PKS (Neubauer et al., 2016). With the function of other enzymes encoded in the clusters, the linear polyketides are subsequently converted into anthroquinone, benzophenone, and finally the xanthone skeleton (Wezeman et al., 2015). The region 13.3 BGC contains homologues of several of these core enzymes and shows high amino acid sequence similarity to corresponding proteins in the reference BGCs. In addition, genes annotated as short-chain dehydrogenase, FAD-binding domain, drug resistance transporter, NAD(P)H-binding, and oxidoreductase activity, also show high similarities to genes in multiple BGCs. However, several other biosynthetic genes in region 13.3 differ from those in known xanthone-related BGCs, suggesting that this cluster may encode previously uncharacterized xanthone-related metabolites. This hypothesis could be further investigated through targeted metabolite isolation, structural characterization, or heterologous expression.

**Griseovulvin analogs.** The cluster in region 15.2 of *Neocucurbitaria* sp. VM-36 is a hybrid of a T1PKS and fungal-Ripp-like BGC. The core PKS gene in this BGC shows 63% identity with the polyketide synthase in BGC0000070 of *Penicillium aethiopicum*. This BGC was previously shown to be involved in the biosynthesis of the antifungal agents griseofulvin, epidechlorogriseofulvin, norlichexanthone, dehydrogriseofulvin, 4-desmethyl griseofulvin, and griseoxanthone B (Chooi et al., 2010; Cacho et al., 2013). The PKS enzyme, GsfA, was demonstrated to use one acetyl-CoA and six malonyl-CoA to form a benzophenone precursor (Cacho et al., 2013). This precursor is converted into a spirocyclic or grisan scaffold by the cytochrome P450 GsfF, which performs the oxidative coupling between orcinol and phloroglucinol rings(Cacho et al., 2013). In contrast, the region 15.2 BGC of *Neocucurbitaria* sp. VM-36 shows similarity primarily to the core PKS and drug resistance transporter genes of the *P. aethiopicum* BGC. The absence of additional enzymes and the presence of a fungal-Ripp-like gene suggests that region 15.2 may encode a related but potentially distinct biosynthetic pathway (Cacho et al., 2013; Bai et al., 2024).

**Benzenediol lactones.** The cluster in region 4.2 of *Neocucurbitaria* sp. VM-36 shows a high degree of similarity to 7 known BGCs, including BGC0000076 (hypothemycin, 66% of genes show similarity), BGC0001245 (lasiodiplodin, 66% of genes show similarity), BGC0002187 (monorden D, 40% of genes show similarity), BGC0000134 (radicicol, 40% of genes show similarity), BGC0001246 (trans-resorcylide, 33% of genes show similarity), BGC0000045 (dehydrocurvularin, 25% of genes show similarity), and BGC0001057 (zearalenone, 18% of genes show similarity). The compounds associated with these reference BGCs are members of the benzenediol lactone family, a type of fungal polyketide metabolite possessing a macrolide core structure fused into a resorcinol aromatic ring. These compounds have been reported to possess several interesting biological activities, such as cytotoxicity, nematicidal properties, inhibition of various kinases, receptor agonists, anti-inflammatory activities, heat shock responses and immune system modulatory activities (Shen et al., 2015).

The core gene in region 4.2 encodes a protein with highly-reducing (HR) PKS, glutathione S-transferase, and non-reducing PKS enzyme activities, sharing 63% identity with the protein AHV78247.1 in the MIBiG database, as well as 79% identity with AHV78243.1, and 70% identity with AHV78245.1 from *Lasiodiplodia theobromae*, respectively. Besides, genes with predicted cytochrome P450, EmrB/QacA drug resistance transporter, and O-methyltransferase functions also display high similarities with corresponding genes in the hypothemycin BGC of ACD39751.1, ACD39756.1, and ACD39755.1, respectively. Additional tailoring genes in the BGC of region 4.2 may contribute to structural diversification of metabolites associated with this BGC. However, the specific metabolites produced by this cluster remain to be experimentally determined.

In conclusion, although several BGCs showed similarity to previously characterized biosynthetic clusters, 23 of the 34 predicted BGCs in *Neocucurbitaria* sp. VM-36 could not be confidently associated with known biosynthetic pathways or metabolites. These uncharacterized clusters represent promising targets for future investigation of the biosynthetic and chemical diversity of this strain, including their potential relevance to natural product discovery.

### 2.2 Note S2. Molecular networking-based annotation of secondary metabolites

The major constituents with high abundance in the crude extracts of *Neocucurbitaria* sp. VM-36 were annotated as putative CJ-21058 analogues in cluster 5 (Figure 7B), which are tetramic acid-type metabolites containing decalin rings. Different amino acids participating in the formation of the tetramic acid moiety and different side chain groups in the decalin rings enrich the diversity of this type of compounds. The highly abundant metabolite *m/z* 388.248 is annotated as the [M + H]^+^ adduct of CJ-21058 (**1**), corresponding to compound **1**, which was subsequently structurally identified after isolation, while *m/z* 387.247 is assigned as an isotope-related feature with a mass difference of -1.001 Da. Although nodes *m/z* 387.993 and *m/z* 388.487 display different precursor masses, their MS2 fragments are the same as those in *m/z* 387.247, indicating that these nodes may correspond to CJ-21058-related isomers. They were all highly abundant in crude extracts of *Neocucurbitaria* sp. VM-36. Here, it is worth noting that stereoselective structural diversity has been previously reported in this type of compounds (Kato et al., 2018). CJ-21058 has six stereoselective positions, which could lead to the formation of isomers with the same planar structures but different configurations. CJ-21058 was firstly reported in 2002 without determined stereoselective positions (Sugie et al., 2002). Interestingly, two new decalin/tetramic acid hybrid fungal metabolites hyalodendrin A (2*R*, 3*S*, 6*R*, 8*S*, 11*S*, 5’*S*) and hyalodendrin B (2*R*, 3*S*, 6*R*, 8*S*, 11*S*, 5’*R*) were reported in 2020 (Sugie et al., 2002; Mao et al., 2020). They show the same planar structure as CJ-21058, and possess the opposite configuration only at the chiral center CH-5’(Sugie et al., 2002; Mao et al., 2020). Therefore, it is highly likely that these high abundant nodes in cluster 5 may share the same planar structures while potentially differing in stereochemical configuration. In addition, other nodes are also predicted according to edge annotations in this cluster. Node **20** (*m/z* 402.264) has one more -CH_2_ group than **1**, and one less -C atom than node **21** (*m/z* 414.264). Node **22** (*m/z* 400.246) has one less -CH_2_ group than **21**, and it does not have a direct relationship with node **20**. According to their MS2 fragments in Table S2, **21** is predicted to be the [M + H]^+^ adduct of (*E*)-3-(hydroxy(1,3,6-trimethyl-2-((1*E*,3*E*)-penta-1,3-dien-1-yl)-1,2,4a,5,6,7,8,8a-octahydronaphthalen-1-yl)methylene)-5-(hydroxymethyl)-1-methylpyrrolidine-2,4-dione or LL-49F233alpha or phomasetin, which have the same planar structure. Node **20** has a side chain group in the decalin ring that differs from **21**, and it is predicted to be (*E*)-3-((2-((*E*)-but-1-en-1-yl)-1,3,6-trimethyl-1,2,4a,5,6,7,8,8a-octahydronaphthalen-1-yl)(hydroxy)methylene)-5-(hydroxymethyl)-1-methylpyrrolidine-2,4-dione, which is a putative new compound. Compared with **21**, node **22** lacks an N-methyl group in the tetramic acid moiety, and is putatively assigned as (*E*)-3-(hydroxy(1,3,6-trimethyl-2-((1*E*,3*E*)-penta-1,3-dien-1-yl)-1,2,4a,5,6,7,8,8a-octahydronaphthalen-1-yl)methylene)-5-(hydroxymethyl)pyrrolidine-2,4-dione or N-demethylated-LL-49F233alpha or N-demethylated-phomasetin. Both node **23** (*m/z* 374.233) and node **24** (*m/z* 374.232), with ions [M + H]^+^, show one less -CH_2_ group than **1**. According to the spectral fragments, the existence of fragments (*m/z* 217.1953 and *m/z* 245.1908) in the node **23** indicate that its side chains in the decalin ring may be the same as those in node **1**, while the absence of fragment (*m/z* 126.0554) reveals that there is no -N-CH_3_ group in the tetramic acid part. Therefore, node **23** is predicted to be (*E*)-3-(hydroxy(1,3,6-trimethyl-2-((*E*)-prop-1-en-1-yl)-1,2,4a,5,6,7,8,8a-octahydronaphthalen-1-yl)methylene)-5-(hydroxymethyl)pyrrolidine-2,4-dione, corresponding to a putative N-demethylated analogue of CJ-21058. On the contrary, node **24** does not have fragments (*m/z* 217.1953, *m/z* 245.1908, and *m/z* 328.2273). Instead, the presence of fragments (*m/z* 126.0554 and *m/z* 144.0657) indicates the absence of -CH_2_ in the decalin ring and the presence of a -N-CH_3_ group in the tetramic acid part. According to the presence of *m/z* 288.1593 and absence of *m/z* 304.1536, the -CH_3_ group attached to position 4 is proposed to be absent and node **24** is putatively assigned as (*E*)-3-((1,6-dimethyl-2-((*E*)-prop-1-en-1-yl)-1,2,4a,5,6,7,8,8a-octahydronaphthalen-1-yl)(hydroxy)methylene)-5-(hydroxymethyl)-1-methylpyrrolidine-2,4-dione, which has a similar proposed planar structure to equisetin. Compared with **1,** nodes **25** (*m/z* 358.238) and **26** (*m/z* 370.238) have lost -CH_2_O and -H_2_O groups, respectively. Node **25** has lost an -O atom and a -C atom compared with node **23** and **26**. The significantly increased fragments with *m/z* 342.2430 and *m/z* 152.0708 in node **26** and *m/z* 340.2264, *m/z* 330.2433 and *m/z* 140.0708 in node **25**, and the sharply reduced fragment *m/z* 170.0813 in both nodes, suggest structural differences in the tetramic acid moiety rather than the decalin moiety. Node **25** and **26** were putatively assigned as two potentially previously uncharacterized metabolites, corresponding to the [M + H]^+^ ions of (*Z*)-3-(hydroxy(1,3,6-trimethyl-2-((*E*)-prop-1-en-1-yl)-1,4,4a,5,6,7,8,8a-octahydronaphthalen-1-yl)methylene)-1-methyl-1,3-dihydro-2H-4l5-pyrrole-2,4-dione and (*E*)-3-(hydroxy(1,3,6-trimethyl-2-((*E*)-prop-1-en-1-yl)-1,2,4a,5,6,7,8,8a-octahydronaphthalen-1-yl)methylene)-1-methyl-5-methylenepyrrolidine-2,4-dione, respectively.

## 3. Supplementary Figures and Tables

### 3.1 Supplementary Figures

### Figure S1. Initial bioactivity screening of the crude EtOAc extract of VM-36 against the Gram-positive indicator bacterium *B. subtilis* 168 using a disk diffusion assay.

### Figure S2. Contigs of *Neocucurbitaria* sp. VM-36 visualized by Bandage.

### Figure S3. Total ion chromatograms recorded in positive ion mode of EtOAc extracts of *Neocucurbitaria* sp. VM-36 grown on PDA solid medium and DPY liquid medium. (A) *Neocucurbitaria* sp. VM-36 grown on the PDA solid medium at 25 ℃ for 28 days; (B) *Neocucurbitaria* sp. VM-36 grown in the DPY liquid medium at 25 ℃ for 30 days, with shaking at 130 rpm.

**Figure S4.** Structural analysis of compound **1**. (A) UV spectrum of compound **1**; (B) Marfey’s result of compound **1** compared with standards *L*-serine and *D*-serine; (C) Experimental ECD of compound **1** and calculated ECD spectra of (2*R*, 3*S*, 6*R*, 8*S*, 11*S*, 5’*S*)-A and (2*R*, 3*S*, 6*R*, 8*S*, 11*S*, 5’*S*)-B, calculated using half-bandwidths of 0.16 and 0.25.

### Figure S5. ^1^H NMR spectrum of compound 1 in CDCl_3_ (400 MHz).

### Figure S6. ^13^C NMR spectrum of compound 1 in CDCl_3_ (100 MHz).

### Figure S7. HSQC spectrum of compound 1 in CDCl_3_.

### Figure S8. HMBC spectrum of compound 1 in CDCl_3_.

### Figure S9. ^1^H-^1^H COSY spectrum of compound 1 in CDCl_3_.

### **Figure S10.** NOESY spectrum of compound **1** in CDCl_3_**.**

### **Figure S11.** TOCSY spectrum of compound **1** in CDCl_3_.

### **Figure S12.** DEPT spectrum of compound **1** in CDCl_3._

### Figure S13. Atomic coordinates for (2*R*, 3*S*, 6*R*, 8*S*, 11*S*, 5’*S*)-A and (2*R*, 3*S*, 6*R*, 8*S*, 11*S*, 5’*S*)-B, obtained through time-dependent density functional theory (TDDFT) at the B3LYP/6-31G(d) level.

### Figure S14. Bacterial viability analysis before (t = 8 h) and after the addition of compound 1, vancomycin or daptomycin and incubation (t = 48 h). The heatmap represents mean values of CFU/mL ± standard deviation (n = 3) with the colour code being based on the mean values.

### Figure S15. *In vitro* hemolytic activity and mammalian cell cytotoxicity assessment of compound 1. (A) Hemolytic activity of compound 1 toward human red blood cells compared with vancomycin and daptomycin. (B) Cytotoxicity of compound 1 in human bronchial epithelial 16HBE14o- cells compared with vancomycin and daptomycin. Cell metabolic activity was assessed using a resazurin assay, and half-maximal inhibitory concentration (IC₅₀) values are shown for the antibacterial compounds. IC₅₀ values were not determined (n.d.) for the positive and negative controls.

### **Figure S16.** Functional genome annotation of *Neocucurbitaria* sp. VM-36. (A) Gene Ontology annotation (Top 50 terms). The size of each dot corresponds to the number of genes enriched in a given term compared to the background gene set. (B) CAZyme analysis and comparison to other *Cucurbitariaceae* species; GHs, glycoside hydrolases; AAs, auxiliary activities; CEs, carbohydrate esterase; GTs, glycosyl transferases; PLs, polysaccharide lyases; CBMs, carbohydrate binding modules. Strains marked with an asterisk (*) represent Type strains.

### Figure S17. Structures of compounds predicted to be produced by *Neocucurbitaria* sp. VM-36 based on antiSMASH analysis.

### Figure S18. Structures of compounds reported to be produced by fungi from the *Cucurbitariaceae* family. See Table S7 for literature related to these compounds.

### 3.2 Supplementary Tables

### Table S1. *Staphylococcus aureus* strains, including clinical isolates and genetically modified strains, used in this study.

| **Bacterial strain** | **Genotype/description** | **Reference** |
| --- | --- | --- |
| **Reference strains** | | |
| ATCC 29213 | (-)/MSSA | - |
| USA300 | USA 300 wild-type/ MRSA | (McDougal et al., 2003) |
| **Clinical MRSA isolates** | | |
| D15 (CA) | Isolate from community associated (CA) outbreak.  More info. available at: https://www.ebi.ac.uk/ena/ ena /view/PRJEB17079 | (Mekonnen et al., 2017) |
| D17 (HA) | Isolate from hospital associated (HA) outbreak.  More info. Available at: https://www.ebi.ac.uk/ena/ ena /view/PRJEB17079 |  |
| **GMO-MRSA strains** | | |
| NE1353 (Aly/Scah) | USA300JE2, transposon in SAUSA300_2579, Em^R^ | (Fey et al., 2013) |
| NE1593 | USA300JE2, transposon in SAUSA300_2482, Em^R^ |  |
| NE1640 | USA300JE2, transposon in SAUSA300_0739, Em^R^ |  |
| NE1649 | USA300JE2, transposon in SAUSA300_0651, Em^R^ |  |
| NE1688 (Sle1) | USA300JE2, transposon in SAUSA300_0438, Em^R^ |  |
| NE1655 | USA300JE2, transposon in SAUSA300_1383, Em^R^ |  |
| NE1035 | USA300JE2, transposon in SAUSA300_2253, Em^R^ |  |
| NE987 (SsaA) | USA300JE2, transposon in SAUSA300_2249, Em^R^ |  |
| NE402 | USA300JE2, transposon in SAUSA300_1140, Em^R^ |  |
| NE973 (Essh) | USA300JE2, transposon in SAUSA300_0277, Em^R^ |  |
| NE24 (LytN) | USA300JE2, transposon in SAUSA300_2503, Em^R^ |  |

### Table S2. Molecular networking-based putative annotation and prediction of secondary metabolites in *Neocucurbitaria* sp. VM-36.

| **No.** | **Metabolite name** | **t_R_ (min)** | **Spectrum ID** | **Cosine score** | **Library class** | **Experi-mental mass (*m*/*z*)** | **Adduct** | **Molecular formula** | **Molecular weight** | **Exact mass (*m*/*z*)** | **MZ Error PPM (ppm)** | **MS^2^ fragment ions (*m*/*z*)** | **Growth medium** | |
| --- | --- | --- | --- | --- | --- | --- | --- | --- | --- | --- | --- | --- | --- | --- |
|  |  |  |  |  |  |  |  |  |  |  |  |  | **PDA** | **DPY** |
| 1 | Compound **1** (predicted as CJ-21058 analogue in molecular networking analysis) | 20.33 | CCMSLIB00011906012 | 0.82 | Gold | 388.2493 | [M+H]^+^ | C_23_H_33_NO_4_ | 387.52 | 388.2478 | -3.86 | 370.2380, 360.2533, 346.2375, 340.2264, 328.1914, 316.1915, 314.1739, 304.1544, 302.1752, 300.1602, 292.1541, 290.1734, 288.1599, 280.1539, 276.1599, 274.1450, 262.1440, 252.1231, 250.1443, 245.1911, 219.1752, 217.1953, 198.0758, 191.1796, 189.1639, 182.0813, 170.0813, 163.1482, 161.1326, 156.0656, 147.1168, 144.0657, 140.0707, 137.1323, 135.1167, 126.0547, 121.1012, 109.1012, 95.0855, 81.0698 | + | + |
| 2 | FruLeuIle | 2.05 | CCMSLIB00005720268 | 0.71 | Bronze | 407.2399 | [M+H]^+^ | C_18_H_34_N_2_O_8_ | 406.48 | 407.2394 | -1.22 | 389.2281, 371.2182, 353.2076, 335.1962, 323.1968, 307.2026, 276.1463, 258.1340, 245.1859, 230.1388, 212.1283, 132.1020, 102.0554, 86.0963 | - | + |
| 3 | DG(16:1/18:2/0:0) | 23.22 | CCMSLIB00010092517 | 0.82 | Gold | 608.5259 | [M+NH_4_]^+^ | C_37_H_66_O_5_ | 590.93 | 608.5245 | -2.30 | 573.4883, 337.2744, 311.2582, 237.2215, 137.1332, 121.1011, 111.1168, 97.1012, 95.0854 | - | + |
| 4 | DG(18:2/0:0/18:2) | 25.08 | CCMSLIB00010070591 | 0.76 | Gold | 634.5416 | [M+NH_4_]^+^ | C_39_H_68_O_5_ | 616.97 | 634.5410 | -0.95 | 599.5033, 337.2740, 263.2373, 189.1641, 175.1481, 165.1280, 151.1122, 147.1169, 137.1326, 133.1013, 127.1116, 121.1013, 111.1168, 99.0803, 97.1012, 93.0699, 83.0855, 57.0702 | - | + |
| 5 | Asp-Leu | 1.62 | CCMSLIB00003137753 | 0.77 | Bronze | 247.1299 | [M+H]^+^ | C_10_H_18_N_2_O_5_ | 246.26 | 247.1288 | -4.45 | 201.1235, 181.0974, 155.1180, 132.1020, 116.0342, 102.0550, 88.0391, 86.0963, 74.0235, 70.0288 | - | + |
| 6 | AEG(o-16:3/15:0) | 24.61 | CCMSLIB00006678159 | 0.75 | Bronze | 535.4732 | [M+H]^+^ | C_34_H_62_O_4_ | 534.87 | 535.4725 | -1.31 | 257.2459, 149.1327, 147.1171, 137.1332, 135.1168, 123.1169, 121.1015, 109.1013, 97.1011, 95.0855, 83.0853, 81.0699, 71.0856, 69.0699, 67.0544, 57.0700 | - | + |
| 7 | 4-[5-[[4-[5-[acetyl(hydroxy)amino]pentylamino]-4-oxobutanoyl]-hydroxyamino]pentylamino]-4-oxobutanoic acid | 18.83 | CCMSLIB00000845585 | 0.84 | Gold | 478.2882 | [M+NH_4_]^+^ | C_20_H_36_N_4_O_8_ | 460.53 | 478.2927 | 9.40 | 337.2738, 306.2797, 263.2365, 245.2262, 216.0642, 163.1483, 149.1327, 147.1164, 137.1324, 135.1171, 121.1014, 109.1012, 95.0855, 83.0853, 81.0698 | + | + |
| 8 | AEG(o-18:4/18:2) | 25.12 | CCMSLIB00006678148 | 0.73 | Bronze | 599.5045 | [M+H]^+^ | C_39_H_66_O_4_ | 598.95 | 599.5040 | -0.83 | 543.4783, 525.4657, 473.4034, 459.3823, 361.2741, 337.2747, 333.2796, 321.2787, 319.2646, 309.2791, 263.2371, 245.2276, 221.1899, 203.1799, 189.1636, 177.1636, 175.1484, 165.1642, 163.1482, 161.1328, 153.1275, 151.1482, 149.1327, 137.1326, 135.1169, 123.1169, 121.1013, 113.0960, 111.1170, 109.1012, 99.0805, 97.1011, 95.0855, 85.0646, 83.0855, 81.0698, 69.0699, 67.0543, 57.0701, 55.0545 | - | + |
| 9 | Riboflavin | 5.79 | CCMSLIB00005884516 | 0.92 | Gold | 377.1467 | [M+H]^+^ | C_17_H_20_N_4_O_6_ | 376.37 | 377.1454 | -3.44 | 359.1352, 341.1257, 243.0878, 200.0821, 135.0648, 117.0545, 99.0440, 75.0440, 73.0283, 69.0335, 61.0286, 57.0338 | + | + |
| 10 | Isousnic acid | 16.70 | CCMSLIB00005719927 | 0.83 | Bronze | 377.1242 | [M+H+CH_3_OH]^+^ | C_18_H_16_O_7_ | 344.32 | 377.1242 | 0 | 345.0982, 327.0874, 317.1031, 303.0871, 285.0754, 275.0919, 261.0769, 251.0917, 235.0982, 233.0816, 219.0662, 85.0285 | + | - |
| 11 | N-[3-[5,8-bis[3-[acetyl(hydroxy)amino]propyl]-14-(hydroxymethyl)-3,6,9,12,15,18-hexaoxo-1,4,7,10,13,16-hexazacyclooctadec-2-yl]propyl]-N-hydroxyacetamide/ desferriferricrocin | 2.80 | CCMSLIB00000848268 | 0.76 | Gold | 718.3377 | [M+H]^+^ | C_28_H_47_N_9_O_13_ | 717.73 | 718.3379 | 0.27 | 546.2548, 528.2414, 471.2210, 402.1983, 374.1669, 356.1574, 345.1785, 317.1454, 314.1433, 303.1650, 299.1366, 285.1560, 275.1345, 272.1255, 230.1141, 190.1179, 188.1032, 173.0922, 145.0973, 131.0815, 114.0549, 86.0600, 70.0650 | - | + |
| 12 | Linoleic acid | 21.10 | CCMSLIB00000567923 | 0.91 | Bronze | 281.2486 | [M+H]^+^ | C_18_H_32_O_2_ | 280.45 | 281.2477 | -3.20 | 263.2372, 221.2270, 195.1591, 193.1591, 181.1591, 179.1433, 169.1221, 167.1435, 165.1275, 153.1277, 139.1119, 129.0909, 125.1327, 115.0753, 111.1169, 97.1012, 85.1012, 57.0702 | + | + |
| 13 | Arg-C18:2 | 15.45 | CCMSLIB00010010944 | 0.82 | Silver | 437.3497 | [M+H]^+^ | C_24_H_44_N_4_O_3_ | 436.64 | 437.3488 | -2.05 | 420.3224, 419.3378, 378.3013, 239.1433, 175.1194, 158.0927, 116.0707, 115.0867, 114.1026, 112.0870, 70.0651 | - | + |
| 14 | 9,10-epoxy-12(*Z*)-octadecenoic acid | 16.28 | CCMSLIB00003134812 | 0.84 | Bronze | 279.2330 | [M+H-H_2_O]^+^ | C_18_H_32_O_3_ | 296.45 | 279.2319 | -3.93 | 261.2215, 209.1542, 195.1380, 181.1589, 167.1435, 165.1276, 163.1121, 153.1276, 151.1483, 137.1326, 123.1169, 113.0961, 99.0805, 97.1012, 85.1012, 71.0855, 57.0702 | - | + |
| 15 | Gly-C18:2 | 20.32 | CCMSLIB00010010949 | 0.80 | Silver | 338.2701 | [M+H]^+^ | C_20_H_35_NO_3_ | 337.50 | 338.2676 | -7.39 | 263.2377, 245.2268, 175.1481, 161.1326, 147.1169, 133.1014, 121.1012, 109.1012, 95.0855, 81.0698 | - | + |
| 16 | 9S-Hydroxy-10*E*,12*Z*,15*Z*-octadecatrienoic acid | 17.10 | CCMSLIB00003135658 | 0.84 | Bronze | 277.2173 | [M+H-H_2_O]^+^ | C_18_H_30_O_3_ | 294.44 | 277.2165 | -2.88 | 277.2164, 259.2060, 235.1698, 231.2105, 221.1531, 217.1953, 207.1383, 189.1665, 189.1276, 181.1220, 175.1485, 153.0910, 149.1328, 145.1012, 135.1170, 123.1170, 109.1012, 95.0855, 93.0699, 69.0699, 67.0543 | + | + |
| 17 | 1,4a-dimethyl-9-oxo-7-propan-2-yl-3,4,10,10a-tetrahydro-2H-phenanthrene-1-carboxylic acid | 17.36 | CCMSLIB00004699026 | 0.86 | Bronze | 315.1966 | [M+H]^+^ | C_20_H_26_O_3_ | 314.43 | 315.1955 | -3.48 | 269.1908, 227.1801, 199.1120, 187.1120, 147.0806, 69.0701 | - | + |
| 18 | Phytosphingosine | 14.71 | CCMSLIB00003134993 | 0.89 | Bronze | 318.3014 | [M+H]^+^ | C_18_H_39_NO_3_ | 317.51 | 318.3002 | -3.77 | 300.2899, 282.2793, 270.2793, 265.2532, 264.2687, 252.2688, 90.0549, 74.0600, 60.0446 | - | + |
| 19 | 1-Linoleoylglycerol | 20.71 | CCMSLIB00003138475 | 0.88 | Bronze | 355.2854 | [M+H]^+^ | C_21_H_38_O_4_ | 354.53 | 355.2847 | -1.97 | 337.2740, 263.2370, 247.2421, 179.1794, 165.1640, 153.1282, 151.1481, 141.1275, 137.1328, 125.1332, 113.0961, 111.1167, 97.1013, 93.0697, 85.0649, 71.0857, 57.0701 | - | + |
| **Putative structures according to molecular network** | | | | | | | | | | | | | |  |
| 20* | (*E*)-3-((2-((*E*)-but-1-en-1-yl)-1,3,6-trimethyl-1,2,4a,5,6,7,8,8a-octahydronaphthalen-1-yl)(hydroxy)methylene)-5-(hydroxymethyl)-1-methylpyrrolidine-2,4-dione | 20.74 | - | - | - | 402.2650 | [M+H]^+^ | C_24_H_35_NO_4_ | 401.55 | 402.2643 | -1.74 | 384.2536, 374.2690, 372.2547, 328.1920, 318.1698, 314.1754, 304.1541, 292.1546, 290.1753, 288.1606, 266.1389, 259.2064, 231.2108, 217.1954, 203.1797, 189.1639, 177.1642, 170.0814, 161.1325, 144.0656, 135.1170, 109.1012, 100.0392, 95.0855, 83.0853 | + | + |
| 21* | (*E*)-3-(hydroxy(1,3,6-trimethyl-2-((1*E*,3*E*)-penta-1,3-dien-1-yl)-1,2,4a,5,6,7,8,8a-octahydronaphthalen-1-yl)methylene)-5-(hydroxymethyl)-1-methylpyrrolidine-2,4-dione/ LL-49F233alpha/ phomasetin | 20.96 | - | - | - | 414.2650 | [M+H]^+^ | C_25_H_35_NO_4_ | 413.56 | 414.2643 | -1.68 | 396.2534, 386.2700, 354.2072, 346.2014, 328.1902, 318.1700, 304.1549, 300.1590, 288.1595, 286.1424, 278.1393, 271.2066, 266.1383, 243.2108, 229.1949, 224.1281, 215.1797, 198.0791, 188.0556, 175.1483, 173.1331, 170.0448, 156.0656, 147.1167, 144.0657, 140.0339, 137.1325, 121.1013, 109.1014, 107.0856, 95.0853, 81.0699, 69.0700 | + | + |
| 22* | (*E*)-3-(hydroxy(1,3,6-trimethyl-2-((1*E*,3*E*)-penta-1,3-dien-1-yl)-1,2,4a,5,6,7,8,8a-octahydronaphthalen-1-yl)methylene)-5-(hydroxymethyl)pyrrolidine-2,4-dione/ N-demethylated-LL-49F233alpha/ N-demethylated- phomasetin | 20.52 | - | - | - | 400.2487 | [M+H]^+^ | C_24_H_33_NO_~~4~~_ | 399.53 | 400.2460 | -6.74 | 382.2382, 372.2541, 340.1917, 332.1856, 314.1768, 290.1384, 288.1589, 274.1441, 271.2061, 264.1235, 243.2107, 215.1797, 189.1642, 175.1486, 173.1332, 156.0295, 147.1171, 142.0501, 138.0186, 137.1325, 130.0502, 121.1013, 109.1012, 107.0856, 95.0855, 81.0699, 69.0700, 67.0544, 60.0445, 55.0545 | - | + |
| 23* | (*E*)-3-(hydroxy(1,3,6-trimethyl-2-((*E*)-prop-1-en-1-yl)-1,2,4a,5,6,7,8,8a-octahydronaphthalen-1-yl)methylene)-5-(hydroxymethyl)pyrrolidine-2,4-dione | 20.03 | - | - | - | 374.2331 | [M+H]^+^ | C_22_H_31_NO_4_ | 373.49 | 374.2327 | -1.06 | 356.2219, 346.2376, 328.2273, 304.1536, 245.1098, 217.1953, 191.1796, 170.0813, 156.0656, 95.0854 | + | + |
| 24* | (*E*)-3-((1,6-dimethyl-2-((*E*)-prop-1-en-1-yl)-1,2,4a,5,6,7,8,8a-octahydronaphthalen-1-yl)(hydroxy)methylene)-5-(hydroxymethyl)-1-methylpyrrolidine-2,4-dione/ equisetin | 19.68 | - | - | - | 374.2331 | [M+H]^+^ | C_22_H_31_NO_4_ | 373.49 | 374.2324 | -1.87 | 356.2224, 346.2379,  288.1584, 252.1229, 170.0814, 144.0657, 126.0554, 95.0856 | + | + |
| 25* | (*Z*)-3-(hydroxy(1,3,6-trimethyl-2-((*E*)-prop-1-en-1-yl)-1,4,4a,5,6,7,8,8a-octahydronaphthalen-1-yl)methylene)-1-methyl-1,3-dihydro-2H-4l5-pyrrole-2,4-dione | 21.27 | - | - | - | 358.2382 | [M+H]^+^ | C_22_H_31_NO_3_ | 357.49 | 358.2376 | -1.67 | 340.2277, 330.2433, 217.1955, 191.1798, 140.0708, 95.0855 | - | + |
| 26* | (*E*)-3-(hydroxy(1,3,6-trimethyl-2-((*E*)-prop-1-en-1-yl)-1,2,4a,5,6,7,8,8a-octahydronaphthalen-1-yl)methylene)-1-methyl-5-methylenepyrrolidine-2,4-dione | 20.55 | - | - | - | 370.2387 | [M+H]^+^ | C_23_H_31_NO_3_ | 369.51 | 370.2381 | -1.62 | 342.2430, 340.2266, 288.1600, 227.1798, 191.1797, 152.0708, 95.0855 | - | + |

### Table S3. Free energies ($\boldsymbol{\Delta}$G) and Boltzmann distribution abundances of conformers for (2*R*, 3*S*, 6*R*, 8*S*, 11*S*, 5’*S*)-A and (2*R*, 3*S*, 6*R*, 8*S*, 11*S*, 5’*S*)-B.

| **Conformers** | **B3LYP/6-31+G(d) Gibbs free energy** | | |
| --- | --- | --- | --- |
|  | **E(TD-HF/TD-DFT)** | $\boldsymbol{\Delta}$**G**$\boldsymbol{\times}$**627.5095 (Kcal/mol)** | **Boltzmann distribution** |
| A-1 | -1251.865612 | 1.327183 | 0.035476 |
| A-2 | -1251.865617 | 1.324045 | 0.035665 |
| A-3 | -1251.865689 | 1.278864 | 0.038493 |
| A-4 | -1251.867721 | 0.003765 | 0.331624 |
| A-5 | -1251.867727 | 0 | 0.333740 |
| A-6 | -1251.867355 | 0.233434 | 0.225003 |
| B-1 | -1251.862270 | 0 | 0.304423 |
| B-2 | -1251.861123 | 0.719753 | 0.090271 |
| B-3 | -1251.861122 | 0.720381 | 0.090175 |
| B-4 | -1251.861123 | 0.719753 | 0.090271 |
| B-5 | -1251.861931 | 0.212726 | 0.212543 |
| B-6 | -1251.861930 | 0.213353 | 0.212317 |

### Table S4. MIC values of compound 1 against ESKAPE strains.

| **Bacterial strain** | **MIC (µg/mL)** |
| --- | --- |
| **Gram negative** |  |
| E. cloacae ATCC 13047 | > 64 |
| K. pneumoniae ATCC 13883 | > 64 |
| A. baumannii ATCC 17978 | > 64 |
| P. aeruginosa ATCC 27853 | > 64 |
| E. coli ATCC 25922 | > 64 |
| **Gram positive** |  |
| E. faecalis ATCC 29212 | 4 |
| S. aureus ATCC 29213 | 1 |
| S. aureus NCTC 8325 | 1 |
| S. aureus Newman | 1 |
| S. aureus HG001 | 2 |

### Table S5. Susceptibility of MRSA strains for vancomycin and daptomycin.

| **Bacterial strain** | **MIC ^(a)^ (µg/mL)** | | **Reference** |
| --- | --- | --- | --- |
|  | **Daptomycin ^(b)^** | **Vancomycin ^(c)^** |  |
| **Clinical MRSA isolates** | | | |
| D15 (CA-MRSA) | 1 | 1 | (Mekonnen et al., 2017) |
| D17 (HA-MRSA) | 1 | 1 |  |
| **Tn mutant MRSA strains** | | | |
| NE1353 (Aly/Scah) | 1 | 1 | (Fey et al., 2013) |
| NE1593 | 1 | 1 |  |
| NE1640 | 1 | 1 |  |
| NE1649 | 1 | 1 |  |
| NE1688 (Sle1) | 2 | 1 |  |
| NE1655 | 1 | 1 |  |
| NE1035 | 0.5 | 1 |  |
| NE987 (SsaA) | 1 | 1 |  |
| NE402 | 1 | 1 |  |
| NE973 (Essh) | 1 | 1 |  |
| NE24 (LytN) | 0.5 | 1 |  |
| **Reference strains** | | | |
| ATCC 29213 (MSSA) | 0.5 | 1 | https://www.atcc.org/ |
| USA300 (MRSA) | 1 | 1 | (McDougal et al., 2003) |

(a) Minimum Inhibitory Concentration determined by broth microdilution.
(b) Breakpoints for daptomycin according to European Committee for Antibacterial Susceptibility Testing: Susceptible: MIC ≤2 µg/mL; Resistant: MIC >2 µg/mL
(c) Breakpoints for vancomycin according to European Committee for Antibacterial Susceptibility Testing: Susceptible: MIC ≤1 µg/mL; Resistant: MIC >2 µg/mL

### Table S6. Biosynthetic gene clusters of *Neocucurbitaria* sp. VM-36 predicted by antiSMASH version 7.0.1.

| **Cluster ID** | **Region** | **Contig** | **Type** | **From (nt)** | **To (nt)** | **Most similar known cluster** | **Genbank** | **Type** | **Similarity** |
| --- | --- | --- | --- | --- | --- | --- | --- | --- | --- |
| 1 | 2.1 | 21 | Fungal-Ripp-like | 61425 | 152320 |  |  |  |  |
| 2 | 2.2 | 21 | NRPS-like | 882440 | 944272 |  |  |  |  |
| 3 | 2.3 | 21 | NRPS | 1287667 | 1352639 |  |  |  |  |
| 4 | 3.1 | 18 | Fungal-Ripp-like | 1044648 | 1132776 |  |  |  |  |
| 5 | 3.2 | 18 | Fungal-Ripp-like | 1616536 | 1707578 |  |  |  |  |
| 6 | 3.3 | 18 | NRPS-like | 1949834 | 2008639 | Choline | CH236925.1 | NRP | 1 |
| 7 | 3.4 | 18 | NRPS | 2381577 | 2450639 |  |  |  |  |
| 8 | 4.1 | 24 | Fungal-Ripp-like | 236535 | 321230 |  |  |  |  |
| 9 | 4.2 | 24 | T1PKS | 2457213 | 2533431 | Hypothemycin | EU520417.1 | Polyketide | 0.66 |
| 10 | 4.3 | 24 | Fungal-Ripp-like | 3165551 | 3256546 |  |  |  |  |
| 11 | 5.1 | 39 | Terpene | 1212913 | 1239972 | Squalestatin S1 | LDZW01000177.1 | Terpene | 0.40 |
| 12 | 5.2 | 39 | Terpene | 1648619 | 1680518 |  |  |  |  |
| 13 | 7.1 | 31 | NRPS | 119867 | 183763 | Metachelin C/ metachelin A/ metachelin A-CE/ metachelin B/ dimerumic acid 11-mannoside/ dimerumic acid | ADNJ02000002.1 | NRP | 0.5 |
| 14 | 7.2 | 31 | Fungal-Ripp-like | 997021 | 1088115 |  |  |  |  |
| 15 | 7.3 | 31 | NRPS, T1PKS | 2001288 | 2073239 | Phyllostictine A/B | KY682688.1 | NRP + Polyketide | 0.2 |
| 16 | 7.4 | 31 | T1PKS | 2492633 | 2559242 | Scytalone/T3HN | KI912112.1 | Polyketide | 0.4 |
| 17 | 8.1 | 7 | Fungal-Ripp-like | 86664 | 177673 |  |  |  |  |
| 18 | 8.2 | 7 | NRP-metallophore, NRPS | 270853 | 356969 |  |  |  |  |
| 19 | 8.3 | 7 | T1PKS | 437365 | 505222 |  |  |  |  |
| 20 | 8.4 | 7 | Terpene | 724153 | 760793 |  |  |  |  |
| 21 | 10.1 | 17 | T1PKS | 525493 | 595142 | (-)-Mellein | KM365454.1 | Polyketide | 1 |
| 22 | 13.1 | 13 | NRPS, T1PKS | 97521 | 169851 | Phomasetin | LC361337.1 | NRP + Polyketide | 0.85 |
| 23 | 13.2 | 13 | Fungal-Ripp-like | 636873 | 727937 |  |  |  |  |
| 24 | 13.3 | 13 | T1PKS | 820674 | 886262 | Secalonic acids | CAGA01000032.1 | Polyketide | 0.37 |
| 25 | 15.1 | 11 | T1PKS | 257292 | 323502 |  |  |  |  |
| 26 | 15.2 | 11 | T1PKS, fungal-Ripp-like | 2976045 | 3120187 | Griseofulvin / Epidechlorogriseofulvin / Norlichexanthone / Dehydrogriseofulvin / 4-desmethylgriseofulvin/ Griseoxanthone B | GU574478.1 | Polyketide: Iterative type I | 0.09 |
| 27 | 17.1 | 19 | NRPS-like | 1170174 | 1233809 |  |  |  |  |
| 28 | 18.1 | 16 | NRPS-like | 238848 | 302100 |  |  |  |  |
| 29 | 20.1 | 28 | T1PKS | 62317 | 136949 |  |  |  |  |
| 30 | 20.2 | 28 | T1PKS | 1167883 | 1219333 | Usnic acid | MG777490.1 | Polyketide | 0.33 |
| 31 | 21.1 | 25 | NRPS | 629575 | 712040 |  |  |  |  |
| 32 | 21.2 | 25 | Fungal-Ripp-like | 1513620 | 1604292 |  |  |  |  |
| 33 | 21.3 | 25 | T1PKS | 1864153 | 1926374 |  |  |  |  |
| 34 | 21.4 | 25 | T1PKS | 3492386 | 3559957 |  |  |  |  |

### Table S7. Compounds reported to be isolated from the Cucurbitariaceae family.

| **No.** | **Name** | **Chemical formula** | **Species** | **Ref.** |
| --- | --- | --- | --- | --- |
| 1 | 8a-hydroxy-spiciferinone | C_14_H_18_O_4_ | *Pyrenochaeta nobilis* | (Oppong-Danquah et al., 2020) |
| 2 | A32390A | C_18_H_24_N_2_O_8_ | *Pyrenochaeta sp.* | (MARCONI et al., 1978) |
| 3 | Pyrenochaetamide A | C_18_H_16_N_2_O_4_ | *Pyrenochaeta sp.* | (Lin et al., 2011) |
| 4 | Pyrenochaetic acid A | C_13_H_14_O_4_ | *Pyrenochaeta terrestris* | (SATO et al., 1981) |
| 5 | Pyrenochaetic acid B | C_13_H_16_O_5_ | *Pyrenochaeta terrestris* | (SATO et al., 1981) |
| 6 | Pyrenochaetic acid C | C_13_H_16_O_4_ | *Pyrenochaeta terrestris* | (SATO et al., 1981) |
| 7 | Pyrenochaetolide A | C_18_H_18_O_7_ | *Pyrenochaeta sp.* | (Lin et al., 2011) |
| 8 | Pyrenochaetolide B | C_10_H_14_O_4_ | *Pyrenochaeta sp.* | (Lin et al., 2011) |
| 9 | Pyrenochaetoxy A | C_18_H_20_O_6_ | *Pyrenochaeta sp.* | (Lin et al., 2011) |
| 10 | Pyrenocine C | C_11_H_14_O_4_ | *Pyrenochaeta terrestris* | (Lin et al., 2011) |
| 11 | Neocucurbol A | C_20_H_28_O_4_ | *Neocucurbitaria unguis-hominis* FS685 | (Hu et al., 2022b) |
| 12 | Neocucurbol B | C_20_H_28_O_4_ | *Neocucurbitaria unguis-hominis* FS685 | (Hu et al., 2022b) |
| 13 | Neocucurbol C | C_20_H_28_O_4_ | *Neocucurbitaria unguis-hominis* FS685 | (Hu et al., 2022b) |
| 14 | Neocucurbol D | C_20_H_28_O_3_ | *Neocucurbitaria unguis-hominis* FS685 | (Hu et al., 2022b) |
| 15 | Neocucurbol E | C_20_H_32_O_2_ | *Neocucurbitaria unguis-hominis* FS685 | (Hu et al., 2022b) |
| 16 | Neocucurbol F | C_20_H_34_O_3_ | *Neocucurbitaria unguis-hominis* FS685 | (Hu et al., 2022b) |
| 17 | Neocucurbol G | C_20_H_32_O_2_ | *Neocucurbitaria unguis-hominis* FS685 | (Hu et al., 2022b) |
| 18 | Neocucurbol H | C_20_H_32_O_3_ | *Neocucurbitaria unguis-hominis* FS685 | (Hu et al., 2022b) |
| 19 | Neocucurbin A | C_20_H_30_O_5_ | *Neocucurbitaria unguis-hominis* FS685 | (Hu et al., 2022a) |
| 20 | Neocucurbin B | C_20_H_30_O_5_ | *Neocucurbitaria unguis-hominis* FS685 | (Hu et al., 2022a) |
| 21 | Neocucurbin C | C_21_H_32_O_4_ | *Neocucurbitaria unguis-hominis* FS685 | (Hu et al., 2022a) |
| 22 | Neocucurbin D | C_20_H_32_O_4_ | *Neocucurbitaria unguis-hominis* FS685 | (Hu et al., 2022a) |
| 23 | Neocucurbin E | C_22_H_34_O_5_ | *Neocucurbitaria unguis-hominis* FS685 | (Hu et al., 2022a) |
| 24 | Neocucurbin F | C_19_H_28_O_5_ | *Neocucurbitaria unguis-hominis* FS685 | (Hu et al., 2022a) |
| 25 | Neocucurbin G | C_19_H_28_O_4_ | *Neocucurbitaria unguis-hominis* FS685 | (Hu et al., 2022a) |
| 26 | Phomalevone A | C_30_H_26_O_10_ | *Phoma sp.* | (Shim et al., 2011) |
| 27 | Phomalevone B | C_30_H_26_O_10_ | *Phoma sp.* | (Shim et al., 2011) |
| 28 | Phomalevone C | C_30_H_24_O_10_ | *Phoma sp.* | (Shim et al., 2011) |
| 29 | Neocucurin A | C_20_H_32_O_2_ | *Neocucurbitaria unguis-hominis* | (Liu et al., 2026) |
| 30 | Neocucurin B | C_20_H_34_O_3_ | *Neocucurbitaria unguis-hominis* | (Liu et al., 2026) |

## 3. Reference

Bai, Y. Bin, Yang, K. M., Zhang, M., Li, Y. X., Zhao, Y., Huang, L. Z., et al. (2024). Synthesis and antifungal activities of novel griseofulvin derivatives as potential anti-phytopathogenic fungi agents. *J. Agric. Food Chem.* 72, 13015–13022. doi: 10.1021/acs.jafc.4c02826

Cacho, R. A., Chooi, Y.-H., Zhou, H., and Tang, Y. (2013). Complexity generation in fungal polyketide biosynthesis: A spirocycle-forming P450 in the concise pathway to the antifungal drug griseofulvin. *ACS Chem. Biol.* 8, 2322–2330. doi: 10.1021/cb400541z

Chooi, Y.-H., Cacho, R., and Tang, Y. (2010). Identification of the viridicatumtoxin and griseofulvin gene clusters from *Penicillium aethiopicum*. *Chem. Biol.* 17, 483–494. doi: 10.1016/j.chembiol.2010.03.015

Cozens, A. L., Yezzi, M. J., Kunzelmann, K., Ohrui, T., Chin, L., Eng, K., et al. (1994). CFTR expression and chloride secretion in polarized immortal human bronchial epithelial cells. *Am. J. Respir. Cell Mol. Biol.* 10, 38–47. doi: 10.1165/ajrcmb.10.1.7507342

Fey, P. D., Endres, J. L., Yajjala, V. K., Widhelm, T. J., Boissy, R. J., Bose, J. L., et al. (2013). A genetic resource for rapid and comprehensive phenotype screening of nonessential *Staphylococcus aureus* genes. *mBio* 4. doi: 10.1128/mBio.00537-12

Hu, J., Zhang, W., Tan, H., Li, S., Gao, X., Liu, Z., et al. (2022a). Neocucurbins A–G, novel macrocyclic diterpenes and their derivatives from *Neocucurbitaria unguis-hominis* FS685. *Org. Biomol. Chem.* 20, 4376–4384. doi: 10.1039/D2OB00585A

Hu, J., Zou, Z., Chen, Y., Li, S., Gao, X., Liu, Z., et al. (2022b). Neocucurbols A–H, phomactin diterpene derivatives from the marine-derived fungus *Neocucurbitaria unguis-hominis* FS685. *J. Nat. Prod.* 85, 1967–1975. doi: 10.1021/acs.jnatprod.2c00249

Kato, N., Nogawa, T., Takita, R., Kinugasa, K., Kanai, M., Uchiyama, M., et al. (2018). Control of the stereochemical course of [4+2] cycloaddition during trans-decalin formation by Fsa2-family enzymes. *Angewandte Chemie* 130, 9902–9906. doi: 10.1002/ange.201805050

Khattab, A. R., and Farag, M. A. (2022). Marine and terrestrial endophytic fungi: a mine of bioactive xanthone compounds, recent progress, limitations, and novel applications. *Crit. Rev. Biotechnol.* 42, 403–430. doi: 10.1080/07388551.2021.1940087

Lin, T., Lin, X., Lu, C., and Shen, Y. (2011). Secondary metabolites of *Pyrenochaeta* sp. B36, an endophytic fungus from *Annona squamosa* L. *Nat. Prod. Res.* 25, 1008–1013. doi: 10.1080/14786419.2010.534997

Liu, H., Hu, J., Chen, Y., Wei, S., Tan, H., and Zhang, W. (2026). Neocucurins A and B, two novel rearranged diterpenes from marine-derived fungus *Neocucurbitaria unguis-hominis*. *Organic Chemistry Frontiers* 13, 3703–3708. doi: 10.1039/D6QO00264A

Mao, Z., Wang, W., Su, R., Gu, G., Liu, Z. L., Lai, D., et al. (2020). Hyalodendrins A and B, new decalin-type tetramic acid larvicides from the endophytic fungus *Hyalodendriella* sp. Ponipodef12. *Molecules* 25. doi: 10.3390/molecules25010114

Marconi, G. G., Molloy, B. B., Nagarajan, R., Martin, J. W., Deeter, J. B., and Occolowitz, J. L. (1978). A32390A, a new biologically active metabolite. II. Isolation and structure. *J. Antibiot. (Tokyo).* 31, 27–32. doi: 10.7164/antibiotics.31.27

Markham, P., Robson, G. D., Bainbridge, B. W., and Trinci, A. P. J. (1993). Choline: Its role in the growth of filamentous fungi and the regulation of mycelial morphology. *FEMS Microbiol. Lett.* 104, 287–300. doi: 10.1111/j.1574-6968.1993.tb05872.x

McDougal, L. K., Steward, C. D., Killgore, G. E., Chaitram, J. M., McAllister, S. K., and Tenover, F. C. (2003). Pulsed-field gel electrophoresis typing of oxacillin-resistant *Staphylococcus aureus* Isolates from the United States: Establishing a national database. *J. Clin. Microbiol.* 41, 5113–5120. doi: 10.1128/JCM.41.11.5113-5120.2003

Mekonnen, S. A., Palma Medina, L. M., Glasner, C., Tsompanidou, E., de Jong, A., Grasso, S., et al. (2017). Signatures of cytoplasmic proteins in the exoproteome distinguish community- and hospital-associated methicillin-resistant *Staphylococcus aureus* USA300 lineages. *Virulence* 8, 891–907. doi: 10.1080/21505594.2017.1325064

Neubauer, L., Dopstadt, J., Humpf, H. U., and Tudzynski, P. (2016). Identification and characterization of the ergochrome gene cluster in the plant pathogenic fungus claviceps purpurea. *Fungal Biol. Biotechnol.* 3, 1–14. doi: 10.1186/s40694-016-0020-z

Oppong-Danquah, E., Budnicka, P., Blümel, M., and Tasdemir, D. (2020). Design of fungal co-cultivation based on comparative metabolomics and bioactivity for discovery of marine fungal agrochemicals. *Mar. Drugs* 18. doi: 10.3390/md18020073

Palma Medina, L. M., Becker, A.-K., Michalik, S., Yedavally, H., Raineri, E. J. M., Hildebrandt, P., et al. (2019). Metabolic cross-talk between human bronchial epithelial cells and internalized *Staphylococcus aureus* as a driver for infection. *Molecular & Cellular Proteomics* 18, 892a–8908. doi: 10.1074/mcp.RA118.001138

SATO, H., KONOMA, K., and SAKAMURA, S. (1981). Three new phytotoxins produced by *Pyrenochaeta terrestris*: Pyrenochaetic acids A, B and C. *Agric. Biol. Chem.* 45, 1675–1679. doi: 10.1271/bbb1961.45.1675

Shen, W., Mao, H., Huang, Q., and Dong, J. (2015). Benzenediol lactones: A class of fungal metabolites with diverse structural features and biological activities. *Eur. J. Med. Chem.* 97, 747–777. doi: 10.1016/j.ejmech.2014.11.067

Shim, S. H., Baltrusaitis, J., Gloer, J. B., and Wicklow, D. T. (2011). Phomalevones A−C: Dimeric and pseudodimeric polyketides from a *Fungicolous hawaiian* isolate of *Phoma* sp. (Cucurbitariaceae). *J. Nat. Prod.* 74, 395–401. doi: 10.1021/np100791b

Sugie, Y., Inagaki, S., Kato, Y., Nishida, H., Pang, C.-H., Saito, T., et al. (2002). CJ-21,058, a new SecA inhibitor isolated from a fungus. doi: 10.7164/antibiotics.55.25

The European Committee on Antimicrobial Susceptibility Testing [EUCAST] (2022). EUCAST reading guide for broth microdilution version 4.0.

Veríssimo, A. C. S., Pinto, D. C. G. A., and Silva, A. M. S. (2022). Marine-derived xanthone from 2010 to 2021: Isolation, bioactivities and total synthesis. *Mar. Drugs* 20. doi: 10.3390/md20060347

Wang, Y., Zhang, B., and Zhai, G. (2016). The effect of incubation conditions on the hemolytic properties of unmodified graphene oxide with various concentrations. *RSC Adv.* 6, 68322–68334. doi: 10.1039/C6RA13607A

Wezeman, T., Bräse, S., and Masters, K. S. (2015). Xanthone dimers: A compound family which is both common and privileged. *Nat. Prod. Rep.* 32, 6–28. doi: 10.1039/c4np00050a

Zehnder, L., Schulzki, T., Goede, J. S., Hayes, J., and Reinhart, W. H. (2008). Erythrocyte storage in hypertonic (SAGM) or isotonic (PAGGSM) conservation medium: influence on cell properties. *Vox Sang.* 95, 280–287. doi: 10.1111/j.1423-0410.2008.01097.x

Zhang, J., Zhang, P., Zeng, G., Wu, G., Qi, L., Chen, G., et al. (2021). Transcriptional differences guided discovery and genetic identification of coprogen and dimerumic acid siderophores in *Metarhizium robertsii*. *Front. Microbiol.* 12. doi: 10.3389/fmicb.2021.783609

Zhang, P., Wang, X., Fan, A., Zheng, Y., Liu, X., Wang, S., et al. (2017). A cryptic pigment biosynthetic pathway uncovered by heterologous expression is essential for conidial development in *Pestalotiopsis fici*. *Mol. Microbiol.* 105, 469–483. doi: 10.1111/mmi.13711

Zhang, P., Zhou, S., Wang, G., An, Z., Liu, X., Li, K., et al. (2019). Two transcription factors cooperatively regulate DHN melanin biosynthesis and development in *Pestalotiopsis fici*. *Mol. Microbiol.* 112, 649–666. doi: 10.1111/mmi.14281

Zhu, S., Yan, Y., Qu, Y., Wang, J., Feng, X., Liu, X., et al. (2021). Role refinement of melanin synthesis genes by gene knockout reveals their functional diversity in *Pyricularia oryzae* strains. *Microbiol. Res.* 242. doi: 10.1016/j.micres.2020.126620
